# It’s the deceiver and the receiver: Individual differences in phishing susceptibility and false positives with item profiling

**DOI:** 10.1371/journal.pone.0205089

**Published:** 2018-10-26

**Authors:** Sabina Kleitman, Marvin K. H. Law, Judy Kay

**Affiliations:** 1 School of Psychology, University of Sydney, Sydney, New South Wales, Australia; 2 Faculty of Engineering & IT, University of Sydney, Sydney, New South Wales, Australia; University of Texas at San Antonio, UNITED STATES

## Abstract

Phishing email is one of the biggest risks to online information security due to its ability to exploit human trust and naivety. Prior research has examined whether some people are more susceptible to phishing than others and what characteristics may predict this susceptibility. Given that there are no standardised measures or methodologies to detect phishing susceptibility, results have conflicted. To address this issue, the current study created a 40-item phishing detection task to measure both cognitive and behavioural indicators of phishing susceptibility and false positives (misjudged genuine email). The task is based on current real-life email stimuli (i.e., phishing and genuine) relevant to the student and general population. Extending previous literature we also designed a methodology for assessing phishing susceptibility by allowing participants to indicate perception of maliciousness of each email type and the actions they would take (keep it, trash it or seek further information). This enabled us to: (1) examine the relationships that psychological variables share with phishing susceptibility and false positives–both captured as consistent tendencies; (2) determine the relationships between perceptions of maliciousness with behavioural outcomes and psychological variables; and (3) determine the relationships between these tendencies and email characteristics. In our study, 150 undergraduate psychology students participated in exchange for partial course credit (98 Females; Mean age = 19.70, SD = 2.27). Participants also completed a comprehensive battery of psychometric tests assessing intelligence, pre- and on-task confidence, Big 6 personality, and familiarity/competence in computing and phishing. Results revealed that people showed distinct and robust tendencies for phishing susceptibility and false positives. A series of regression analyses looking at the accuracy of both phishing and false positives detection revealed that human-centred variables accounted for a good degree of variance in phishing susceptibility (about 54%), with perceptions of maliciousness, intelligence, knowledge of phishing, and on-task confidence contributing significantly, directly and/or indirectly via perception of maliciousness. A regression model looking at discriminating false positives has also shown that human-centred variables accounted for a reasonable degree of variance (41%), with perceptions of maliciousness, intelligence and on-task confidence contributing significantly, directly and/or indirectly via perception of maliciousness. Furthermore, the characteristics of the most effective phishing and misjudged genuine email items were profiled. Based on our findings, we suggest that future research should investigate these significant variables in more detail. We also recommend that future research should capture consistent response tendencies to determine vulnerability to phishing and false positives (rather than a one off response to a single email), and use the collection of the most current phishing email obtained from relevant sources to the population. It is important to capture perceptions of maliciousness of email because it is a key predictor of the action taken on the email. It directly predicts accuracy detection of phishing and genuine email, as well as mediating the relationships between some other predictors whose role would have been overlooked if the perceptions were not captured. The study provides the framework of human-centred variables which predict phishing and false positive susceptibility as well as the characteristics of email which most deceive people.

## Introduction

With improving technology, storing and distributing information has never been easier. As a consequence, new ways of exploiting and obtaining information illegally have also developed. Online phishing is a particularly dangerous means of obtaining confidential information and is defined as “a form of deception in which an attacker attempts to fraudulently acquire sensitive information from a victim by impersonating a trustworthy entity” ([1], p 1). As opposed to other deceitful information-gathering methods (for example, following someone into a secure location; talking to someone with the intent of extracting classified information), phishing is only conducted online. Commonly orchestrated through email, phishing relies on exploiting human trust while bypassing email software detection systems. It exploits what is known as ‘Social Engineering’, where individuals are manipulated into aiding the deceivers, either through actions helpful to the deceiver or by providing confidential information [2].

Phishing has become a global security issue [3]. According to Verizon’s 2017 Data Breach Investigations Report, almost all successful phishing attacks in 2016 were followed with the installation of malware, with 66% of all malware being installed through email attachments. While phishing attacks have targeted the general public, more specific targets such as banks, defence organisations and private companies have being identified as particularly attractive, as these organisations have access to the extensive variety of data they collect and hold, including personally identifiable information, confidential information and financial data [4].

Higher education institutions have also recently become targets of various phishing attempts. In the UK, Duo Security found that 72% of universities which had responded to a Freedom of Information (FoI) request had reported falling for a phishing attack. Furthermore, both high-profile and small universities in the US have reported falling victim to phishing, with a public service announcement from the FBI indicating college students to be specifically targeted using phishing scams promising employment. In accordance with the growing risk of phishing, universities have been pressured to increase phishing awareness for students, with calls coming from institutions such as the UK’s fraud and cybercrime centre, Action Fraud, and the city of London police.

Human error has been suggested to be the weakest link in most secure systems [5]. Existing literature repeatedly identifies peoples’ poor capacities for detecting online phishing, with more than 90% of individuals falling victim to some form of phishing [6]. Even when primed to detect phishing, participants failed to detect 47% of phishing stimuli, and spent little time focussing on security indicators [7]. Unfortunately, despite the prominence of human risk, research has shown that the efficacy of phishing detection software such as security indicators and toolbars are largely limited. It has been shown that over time people increasingly disregard warnings from security software [8–9]. Thus, the limitation of external security systems highlights the need to investigate human factors. Specifically, why people are unable to detect phishing email, referred here as susceptibility to phishing, and whether some people are more susceptible than others.

In this study, we focus on profiling the individual characteristics of higher education students to be vulnerable to phishing. We will also profile the email characteristics that are relevant to targeting students, as well as general population.

### Individual differences in phishing susceptibility

A substantial amount of research has examined relationships between susceptibility to phishing and other individual characteristics [10–12]. However, these results often conflict. For instance, one of the Big 5 personality factor, Openness, correlated *positively* with accuracy in detecting phishing email in one study [13] but *negatively* in another [14]. Despite robust relationships between some variables and phishing susceptibility—gender, trust and attention to phishing stimuli (e.g. [6, 13, 15, 16])—the vast majority of findings are inconsistent within the literature. This is a serious problem for providing foundations that can inform the development of mitigation strategies to phishing susceptibility.

This inconsistency may be due to the use of tasks which are largely unsuitable for examining *robust tendencies* in phishing susceptibility and, thus, its relationships to individual differences variables. This is because, a large, internally consistent distribution of scores is required to reliably examine relationships between individual difference variables [17]. However, with few notable exceptions (see below), studies often limit their distribution of accuracy scores in phishing detection by employing a few phishing email items or by using a small sample of study participants (e.g. [13, 18, 19]). These reduce the validation of the relationships between phishing susceptibility and other variables. Furthermore, within the individual differences literature, study designs often employ a comprehensive set of measures to control for relevant variables when examining possible relationships (e.g. [20]). Consequently, studies examining individual differences, with large samples and email items, as well as a sufficiently comprehensive set of relevant psychological and control variable measures are necessary.

The current study aims to examine the relationship between phishing susceptibility and individual difference characteristics while avoiding the limitations of having few email items by creating and implementing a 40-item email detection task (see Method Section for a detailed description). Within the task, rather than a simple phishing/non-phishing dichotomy, we implemented a novel decision-making procedure which is similar to approaches taken in research on phishing susceptibility [17, 21, 22]. That is, instead of asking people to simply classify the email as being genuine or phishing, we captured peoples’ perception of the degree of maliciousness of each stimulus (0 to 100, with 0 being not malicious at all to 100 being definitely malicious). Perceived maliciousness is an important indication of whether or not a person will keep an email—if the email seems suspicious and perceived maliciousness is high, this feeling should prevent the person from keeping the email. Similarly, if perceived maliciousness is low, the person is more likely to keep the email. Thus, we believe perceived maliciousness facilitates different types of behavioural decisions. In this research, we captured three different decisions by asking participants to indicate the behaviour they decided to apply to the email—keep, trash or seek more information. This last behaviour was included as a reflection of real-life contexts, where people can seek additional information on an email to determine maliciousness (hover over the link, approach their IT specialists, consult relevant anti-phishing websites). Additionally, people may not fall for a phishing email due to reasons other than perceived maliciousness, e.g. a lack of interest [21]. By measuring both perceived maliciousness and behaviour for each phishing email, we are able to investigate their unique relationships with phishing susceptibility. Thus, we predict that perceived maliciousness will act as a key predictor of the behavioural decision.

In addition to the central focus of phishing susceptibility (defined here as the inability to detect phishing email), the present study also separately examined how people approached *genuine* email. This is because we hypothesised that people may approach phishing and genuine email differently. To examine this, we assessed their detection accuracy. If they indicated they would trash a genuine mail item, this was labelled here as a false positive. The other two behaviours, keep or seek more information, were treated as correct. As with phishing email, we expect that perceived maliciousness will predict the accuracy of detecting genuine email but in the opposite direction. That is, the higher the perceived maliciousness, the more likely the genuine email would be trashed and classified as a false positive.

Given the important role that perceived maliciousness plays in facilitating a particular behaviour, this study extends previous research by investigating the individual differences and email characteristics that predict this perception. We will also use perceived maliciousness to predict the accuracy of detection for phishing and genuine email. By doing so, we extend previous research by looking at the possible indirect predictions that individual characteristics may have on phishing susceptibility and false positives via their relationships with perceived maliciousness.

Finally, this study also expands on the current body of literature by examining variables which have rarely or never been investigated with phishing (i.e. intelligence, confidence, and honesty/propriety) in conjunction with variables commonly studied (e.g., knowledge of computers and phishing, age, gender, five personality dimensions). These neglected variables, intelligence, confidence, and honesty/propriety, have potentially important implications for designing counter-measures that are intended to increase the effective detection of phishing email [21–22]. These variables are reviewed in the next sections.

#### Intelligence

Current research on intelligence often follows the Cattell-Horn-Carroll (CHC) Model of Intelligence. This model contains an overarching intelligence factor, ‘g’, which is separated into distinct broad abilities [23]. Within these broad abilities, academic performance has been found to most strongly relate to fluid intelligence (Gf), the capacity for problem solving, and crystallised intelligence (Gc), the capacity for using learnt knowledge [24]. Similarly, in the domain of decision-making, intelligence has been shown to relate strongly to general decision-making [25]. In particular, the broad ability of numeracy has been found to be the best single predictor of individual differences in general decision-making skill [25]. Although Hong et al. [6] measured intelligence within their experiment, there was no indication that they had examined intelligence in relation to phishing susceptibility. Furthermore, Vishwanath, Harrison, & Ng [26]’s research focused on two modes of cognitive-information processing (systematic and heuristics) instead of intelligence as a construct. Nevertheless, Parsons, McCormac, Pattinson, Butavicius, and Jerram [27] found more educated individuals to be less susceptible to phishing when informed about the phishing element in their study.

To our knowledge, the current study is the first to examine a relationship between scores from an actual measure of intelligence and phishing and false positives susceptibility. It is important to study the relationship between deception abilities and intelligence, as greater cognitive capacities (especially captured by Fluid intelligence and verbal reasoning measures) may enhance people’s abilities to sustain their attention and cognitive effort, and process stimuli more efficiently. It is also important to control for verbal comprehension captured by Crystallised intelligence measures. This study employed a mixed measure of Fluid and Crystalized intelligence which, together, capture cognitive ability. It is hypothesised that greater intelligence (here verbal reasoning) predicts both lower phishing and false positives susceptibility.

#### Confidence

Few studies within the phishing literature have examined the relationship between confidence (within phishing tasks and as a broad trait; see [28] for a review) and phishing susceptibility. Studies have found that participants have great confidence in their decisions when filtering phishing and genuine email [11, 29]. When Wang, Li and Rao [30] examined the relationship between on-task confidence and accuracy on a phishing detection task, they found that on-task confidence predicted phishing detection accuracy even when controlling for self-efficacy. Furthermore, Canfield et al. [21] recently found that participants’ ability to discriminate phishing email is related to confidence, and such a result is expected, aligning with other areas of research where correlations between confidence and accuracy have been positive and significant (e.g. [28]). Thus, it is expected that phishing detection task confidence will correlate negatively with both phishing susceptibility and frequency of false positives. It is unclear, however, whether the same relationships would exist when relating phishing susceptibility and false positives with confidence judgments provided on a different cognitive test, here, a measure of intelligence. If such a relationship exists, it would suggest that more confident people (people high on the trait of Confidence, see [28] for a review) are in general less susceptible to phishing email. However, if confidence captured within the phishing test only shares a relationship with phishing susceptibility, it would signal domain-specificity of such a relationship, possibly informing the design of the type of training required.

#### Personality

Several studies have examined the relationship between the Big 5 personality traits and phishing susceptibility. However, their findings appear to conflict. For example, Alseadoon et al. [13] found higher agreeableness, openness and extraversion to correlate with greater phishing susceptibility whilst Halevi, Memon, and Nov [14] found only conscientiousness to correlate with greater phishing susceptibility. These inconsistent findings may be attributed to the common use of only one phishing stimulus to examine individual differences in phishing detection within the literature (e.g. [13]). However, Pattinson, Jerram, Parsons, McCormac, and Butavicius [22] examined personality and phishing detection with a scenario-based phishing detection measure, as well as with a large number of phishing stimuli (50 items). With a sample who were informed of the phishing task, the study (N = 58) found that correlations between personality and phishing susceptibility were low and ranged between -.11 to .18. These low correlations were also found between personality and frequency of false positives, ranging between -.24 and .15. Furthermore, most of these correlations were not statistically significant (especially given a small sample size). Extending this finding, the current study employed a Big-6 personality measure which is an alternative personality factor model that includes an additional Honesty-Humility factor [31]. Following [22], it is expected that there are weak relationships between personality and phishing susceptibility or with frequency of false positives.

#### Knowledge of computers and phishing

Although many studies have examined the role of computer and phishing-specific knowledge on phishing susceptibility, no consistent relationship has been found (e.g. [7, 13, 19, 32]). The varied findings in the literature may be due to the large range of different questions used between studies to assess either knowledge of computers (e.g. knowledge of computer risks, experience with email), or knowledge of phishing (e.g. past phishing experiences, knowledge of phishing and lock icons) ([10, 13, 33]). The current study used an approach which included an *objective measure* by asking people to provide a definition of phishing (similar to [10] and [33]) in addition to *self-report* measures of knowledge of computers and phishing (e.g., Familiarity and Knowledge of Computers and Phishing, Risk Profile Questionnaire) (similar to those measured in [15]). Within Downs et al. [10], those with greater knowledge of phishing and computers (specifically regarding lock icons) were less likely to fall for phishing email. Although, Vishwanath et al. [15] did not examine the direct relationship between self-reported knowledge of computers and phishing, Wright and Marett [34] found that extensive security training (8- weeks) and greater computer self-efficacy predicted lower phishing instances. Furthermore, Rhee, Kim and Ryu [35] found a significant positive correlation between self-efficacy about computers and more secure online behaviour. The same outcomes are expected in this research, with both objective and self-reported measures of knowledge in phishing and computers predicting less susceptibility to phishing email and false positives.

#### Age

The common use of university student samples in the literature has made it difficult to determine the relationship between age and phishing susceptibility [11, 36]. Most studies have found no effect of age on phishing susceptibility [11, 37]. However, Sheng et al. [33] found 18–25 year olds to be more susceptible to phishing, though this was largely mediated by other factors such as exposure to prior training, risk taking behaviour, level of education and technical expertise. The current study uses a university student sample, thus, limiting the distribution of age to this most susceptible group. The question examined in this study is whether there are still any age differences within this group.

#### Gender

Studies in the literature consistently show women are more susceptible to phishing stimuli than men (e.g. [32, 37]). Although this result is partially mediated by technical knowledge and training [33], other variables may explain this gender difference and further investigation is needed. Following previous research, the current study expects women to have higher phishing susceptibility and frequency of false positives for genuine email than men. We also expect that women tend to be more likely to perceive maliciousness in both types of email, due to their risk-aversive tendencies [38].

### Influences of email characteristics on phishing susceptibility

In addition to human characteristics, it is also important to examine how email characteristics may affect phishing susceptibility. Although email detection software can rely on machine learning to accurately detect most phishing attempts, weaknesses in these systems eventually result in phishing attacks falling through to people’s inbox [39–40]. Subsequently, the lack of knowledge in phishing targets often result in successful phishing and theft of important information [3]. To reduce the risk of such an occurrence, it is important to examine which characteristics in email are most trust and suspicion-inducing. Ultimately, this would inform both phishing detection training and ways for legitimate sources to structure their email to avoid false positives.

Existing research into phishing characteristics has provided researchers with the means of recommending counter-phishing strategies such as using the HTTPS text in the address bar as an indicator for legitimacy and to be suspicious of email asking for private information [41–42]. Within the literature, the majority of email characteristics which are highlighted by researchers as useful for phishing detection can be categorised into those that induce greater trust (e.g. official domains such as edu) and those that induce greater suspicion (e.g. misspellings and grammatical errors) [37; 41]. However, phishing attacks can also be categorised through neutral characteristics such as the email’s intended target group [12]. The email’s target group often varies depending on the motivation behind the phishing attack, with more specific targeting often resulting in greater success of the attack [26, 43]. Thus, aligning with previous research, the current paper examines email characteristics, separating them into neutral, suspicion-inducing (i.e. known phishing email characteristics) and trust-inducing characteristics, as shown in Table 1 below.

**Table 1:** Characteristics of email used in the phishing detection task.

Email Characteristics	Description	Distribution within Stimuli
*Neutral Email Characteristics*		
Email Word Length	How many words are in the email?	Mean = 138.75, SD = 171.19, Range = 12–1075.
Target	Who is the email is intended for? (Generic is for the general population, i.e. [No mentions of any group], Loosely-Targeted is for a type of population e.g. “The Library of Alexandria is contacting you and other great researchers in the world”, Spear-phishing is intended for a specific group e.g. “Sydney University Students Needed”	Generic- 16, Loosely-targeted– 14,Spear phishing– 10
Contact Method	How is the email asking the sender to respond? Email Reply–“If interested, revert back to my personal email: ricclf01@aol.co.uk for more details”, Hyperlink–“Click on this link Verify Automatically”	Email Reply– 12, File Attachment– 3, Hyperlink– 23, Hyperlink + Email Reply– 1, None– 1
Number of URL	The number of URL links contained within the email	Mean = 1.55, SD = 1.81, Range = 0–7
*Known Phishing Email Characteristics*		
Asks for Confidential Information	Asks for confidential information from their victim e.g., “Verify your email address to have full access to the document!!”	Yes- 9, No- 31
Misspellings/Grammatical Errors	Contains mistakes in spelling and grammar within the email, e.g. “John McKinnon recently send you confidential Dropbox file”	Yes- 19, No- 21
Pressure	Incites pressure for victims to respond without thinking clearly, e.g., “Your mail box is cramped with unsolicited mails and will be suspended if you don’t prove it is not used for fraudulent acts.”	Yes-19, No- 21
Vague Recipient	Addresses victim vaguely (e.g. Sir) rather than by name, e.g. “Hi friend”	Yes– 30, No– 10
Suspicious Email Domain	Contains email domains which are easily obtained and/or do not suit the sender’s character, e.g. Super53@lista.pitt.edu for an academic library	Yes-1, No- 39
Suspicious URL	Contains URLs which contain non-letter characters, are long and/or have a different URL domain than the official one. E.g. http://ova-maintenance.sitey.me/ for a University of Sydney IT Service	Yes-8, No-32
*Email Characteristics which Induce Trust*		
Official Email Domain	Contains email domains which are analogous to the sender’s character and are difficult to obtain (e.g. rose.kendrik@acu.edu.au)	Yes- 9, No- 31
Official URL	Contains URLs which link to official email and are difficult to obtain, e.g. “http://www.education.gov.au/help”	Yes- 4, No -36
Use of URLs with HTTPS	Contains URLs with HTTPS indicates that the information processed by it are encrypted, although the site may still be a phishing scam, e.g. “https://www.surveymonkey.com/r/3D385CB”	Yes- 6, No -34

Based on past literature, it is expected that there would be an effect of the target victim, such that the more specific the target group, the more likely participants are to keep email and less likely to choose trashing behaviour. We expect trust-inducing email characteristics to influence participants into keeping behaviour whilst suspicion-inducing email characteristics would influence participants into trashing behaviour. Furthermore, it is expected that these characteristics would have an additive effect on behaviour, such that the more trust/suspicion inducing characteristics an email has, the more likely participants would be to respond with the associated behaviour (e.g. more trust-inducing characteristics would increase the likelihood of keeping behaviour and reduce the likelihood of trashing behaviour).

### Aims and hypotheses

This study has three overarching aims. The first aim of this study is to determine the characteristics surrounding individuals and stimuli that predict *phishing susceptibility*. Secondly, we will examine susceptibility to *false positives by* also collecting information about how people responded to genuine email. The third aim is to scrutinise the characteristics of the most effective maliciousand the most phishing-like genuine email. To aid and streamline the interpretations we operationalised both phishing susceptibility and false positives as Phishing and Genuine Detection Accuracy variables, respectively. The relevant hypotheses are presented below.

Aim 1. Phishing Susceptibility: a. Phishing Detection Accuracy and b. Perceived Maliciousness of Phishing Email.

To determine the relationships between *phishing detection accuracy* and different individual differences variables, t-tests and correlations were conducted first, followed by a regression analysis to examine the following hypotheses:

1. Intelligence will predict *positively* (a) detection accuracy of phishing email and (b) perception of maliciousness of phishing email;
2. On-task confidence for phishing email will predict *positively* (a) detection accuracy of phishing email and (b) perception of maliciousness of phishing email;
3. Both objective (accuracy of phishing definition) and subjective (e.g. reported competence in phishing detection and awareness of padlock icon) measures of knowledge of phishing and computers will predict *positively* (a) phishing detection accuracy and (b) perception of maliciousness of phishing email;
4. Perception of email maliciousness will strongly and *positively* predict phishing detection accuracy.
  Aim 2. False Positives: a. Genuine Detection Accuracy and b. Perceived Maliciousness of Genuine Email.
  To determine the relationships between accuracy in detecting genuine email and different individual difference variables, t-tests and correlations were conducted first, followed by regression analyses to examine the following hypotheses:
5. Intelligence will predict (a) *positively* detection accuracy of genuine email and (b) *negatively* perception of maliciousness of genuine email;
6. On-task confidence for genuine email will predict (a) *positively* detection accuracy of genuine email and (b) *negatively* perception of maliciousness of genuine email;
7. Both objective and subjective measures of knowledge of phishing/computers will predict (a) *positively* detection accuracy of genuine email and (b) *negatively* perception of maliciousness of genuine email;
8. Perception of maliciousness of genuine email will predict strongly and *negatively* detection accuracy of genuine email (more false positives).
  Replicating previous findings, we expect:
9. Women will be more susceptible to phishing and to false positives than men;
10. Personality traits will share small and possibly negligible relationships with (a) detection accuracy of phishing and genuine email and (b) perception of maliciousness of phishing and genuine email.

Aim 3. The third aim of this study is to determine what characteristics make a malicious email most susceptible to keeping. Four phishing email items with the highest frequency of keeping and four genuine email items with the highest frequency of trashing were profiled.

## Methods

### Participants

Participants in the current study consisted of 150 undergraduate psychology students (Female = 98 [65.3%], M = 19.70, SD = 2.27, age range = 17–37) from the University of Sydney and they participated in exchange for course credit. Although the current research only recruited officially university-enrolled undergraduate students as participants with an advertised minimum age of 18, there were 4 students below the age of 18 at the time of testing. Given that participation in research was optional and a part of university requirements (in exchange for course credit with an alternative being an assignment), we allowed these students to participate so as to not limit their educational options. There were some missing data for only four participants. One participant entered the current date of testing as their date of birth. Another participant did not complete the Phishing Detection Task. A participant’s Phishing Detection Task data was removed from further analyses as they had ignored the instructions and had chosen the same action for every item irrespective of the item’s content. Lastly, one participant’s phishing definition score was removed as their definition had been pasted from a web-site. While this may reflect some knowledge, from even conducting the search and reading of the definition, it may well not reflect the real knowledge of the participant. All participants completed the measures online in their own time. Ethics approval for the current study was obtained from the Human Research Ethics Committee at the University of Sydney with Project Number: 2016/387.

### Measures

#### Phishing detection task

The Phishing Detection Task contains twenty phishing and genuine email each (similar to [21] and [22]) with a behavioural response component being used to determine phishing detection accuracy.

**Fig 1:**
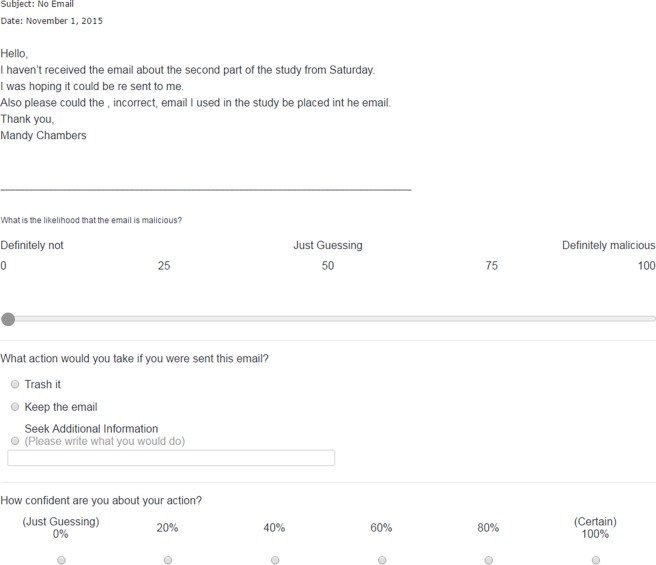
A sample genuine item from the phishing detection task.

**Fig 2:**
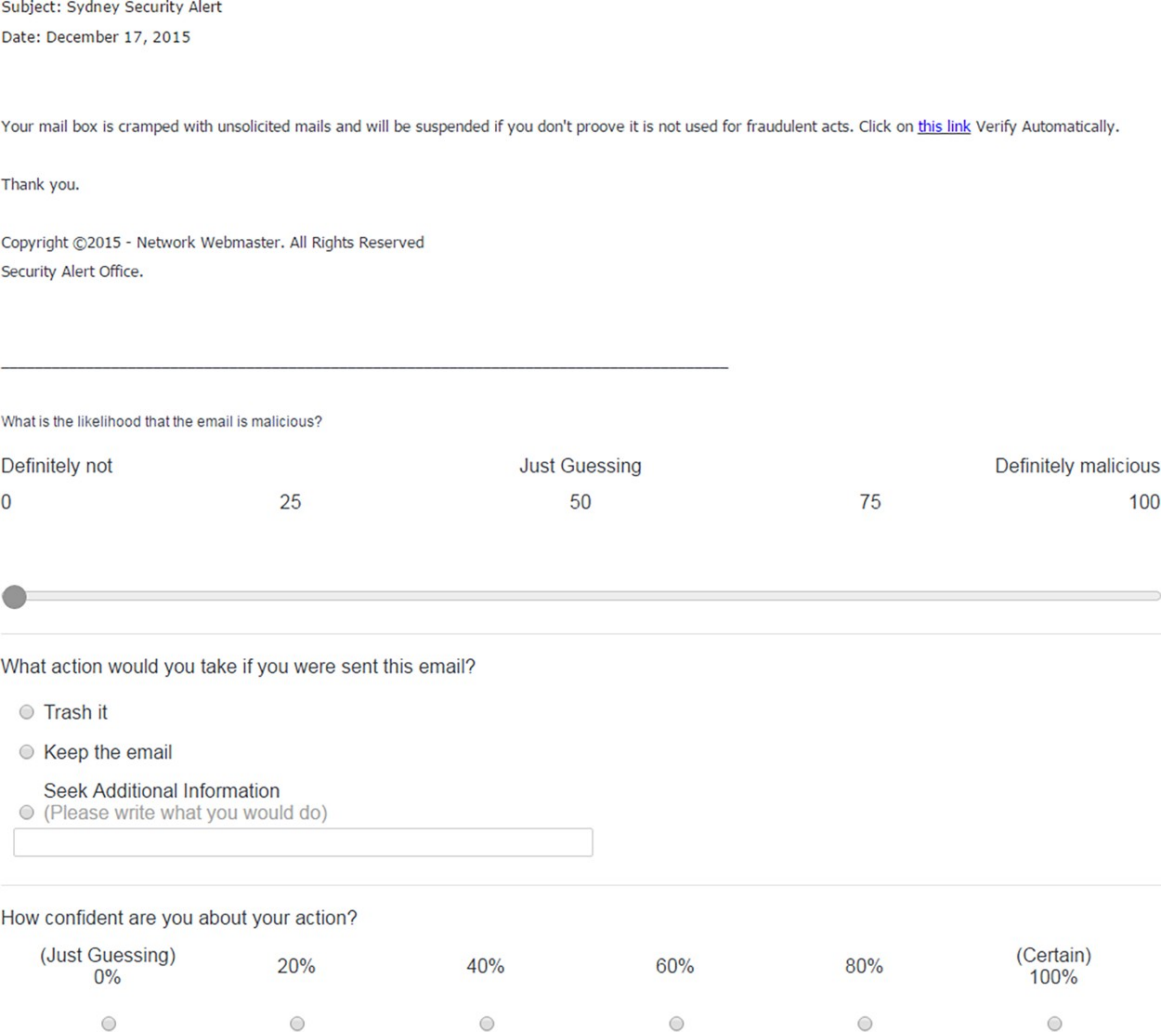
A sample phishing email item from the phishing detection task.

Email items were oriented to public and student populations and presented to the participants in a randomised order. To resemble real-life scenarios, all email items were obtained from real and current sources, including public websites dedicated to anti-phishing awareness (e.g. Cornell University’s Anti-Phishing Database), those recently received by the current authors (in 6–8 months prior to conducting this research) and sourced from the University of Sydney Information and Communications Technology (ICT) services. It has been established that there are differences between phishing email in both email characteristics (e.g. specific vs. general recipient) and the capacity to deceive recipients [12, 37]. To reflect the broad range of phishing and genuine email students could receive, email varied systematically on the characteristics shown in Table 1. However, few stimuli contained certain email characteristics e.g. only one email contained a suspicious email domain. Example items can be seen in Figs 1 and 2 below.

Within the task, the email stimuli were adapted to a psychology post-graduate context, and participants were told that they were filtering email for a PhD student. As participants were not filtering for themselves, reasons for keeping/trashing the email other than determining the email’s authenticity were removed. To reflect the PhD context, references to the target were changed to match the student, i.e. name and email. Stimuli were also altered marginally with fictional names/email addresses/URLs to remove any privacy or phishing risks. All other content mirrored the original email to retain authenticity. To help with understanding the task, an accurate definition for phishing was provided to participants (this was only after the task in which participants gave their own definition). Participants were shown each email statically, and thus, interactive actions for evaluating legitimacy such as hovering over links were not possible. Instead, specified in the instructions, participants were provided the option to seek further information regarding the email if they were unable to classify the email with the information provided and then relay what information they would seek, e.g. hovering over the link, a search through known ‘anti-phishing’ websites and/or to pursue some other options.

In decision-making paradigms, people have to cognitively process cues related to an event before they act or make a behavioural decision [44]. Thus, to capture phishing susceptibility both cognitively and behaviourally, participants were asked to rate how malicious each email appeared (0 to 100, with 0 being not malicious at all to 100 being definitely malicious) as well as what behaviour they would decide on for the email (keep, trash or seek more information). Using these two measures, three indications of phishing susceptibility were computed; average perceptions of maliciousness (for phishing and genuine email separately), frequencies of the three behaviours and email detection accuracy. Email detection accuracy was coded with correct responses being trashing or seeking more information for phishing email; and keeping or seeking more information for genuine email. Seeking more information was coded as correct for both phishing and genuine email to reflect the correct behaviour in real-life situations and its benefit to more accurate phishing detection [45]. A false positive was a legitimate email message that was incorrectly identified as phishing (i.e. trashed). Table 2 summarises the metrics used in the Phishing Detection Task.

**Table 2:** Metrics used in the phishing detection task.

Phishing email	Genuine Email
***Detection Accuracy*: *Phishing*** Actions: Trash or seek more information	***False Positive*** Actions: Trash
***Phishing*** Actions: Keep	***Detection Accuracy*: *Genuine*** Actions: Keep or seek more information

To capture on-task confidence, participants were also asked how confident they were in their behavioural decision about the email using a six point 0 to 100 scale, with 0 being not confident at all and 100 being absolutely confident. Six points of reference (0%, 20%, 40%, 60%, 80% and 100%) were used on the scale to ensure that all respondents interpret the scale in the same way. Overall accuracy scores for email detection and average rating scores for both confidence and perceived maliciousness were calculated.

Although the current measure was most similar to [22], there are several fundamental differences. Firstly, our measure did not contain the option to follow up an email, or to block the sender. Following up on an email is ambiguous, and can be construed as different behaviours such as seeking more information or opening the links in the email at a later time. Furthermore, deleting the email and blocking the sender was not provided as an option as phishing email is often sent from different email addresses. In fact, blocking the sender may be a more appropriate response to spam email rather than phishing email. We provided a third option to seek further information which we believe is more consistent with university student behaviour for email. The scoring methodology is also different compared with [22] due to the changes in options, as there is only one behavioural option to keep the email against [22]’s two keeping email options of leaving the email in the inbox only and leaving the email in the inbox and following up later. Similar to [27], all scores were calculated separately for phishing and genuine email.

#### Individual difference variables

**Intelligence: Esoteric analogies test (EAT; from the Gf/Gc quickie battery, [46])**

To index the cognitive ability of participants, the Esoteric Analogies Test was used. The 24-item measure captures both fluid and crystallised intelligence ([46]), with each item consisting of verbal analogies. Participants must determine which of the four multiple-choice answers relates most to a specific word in the same manner as the first pair of words. A sample item is: FORE is to AFT as BOW is to: STERN [STERN]; DECK; BOAT; ARROW. Accuracy scores were calculated by the percentage of items correct with higher scores indicating greater fluid and crystallised intelligence. Previous studies have shown good reliability, Cronbach’s α = .69-.72 [20, 47].

#### Confidence

Confidence measures were taken for the EAT and Phishing Detection Task. Cronbach’s reliability estimates for confidence in the EAT have been found to be higher than .80 (e.g., [20, 47, 48]), with the current study having a Cronbach’s alpha of .88. Cronbach’s reliability for PDT confidence for phishing and genuine email were .93 and .94 respectively, which is consistent with typically high reliability estimates for confidence judgments (see [28] for a review).

#### Big 6 personality inventory [49]

The 25-item Big 6 Personality Inventory measures, Agreeableness (e.g. I am inclined to forgive others), Conscientiousness (e.g. I like order), Extraversion (e.g. I laugh a lot), Honesty/Propriety (e.g. I would never take things that are not mine), Resilience (e.g. I rarely worry), and Originality/Intellect (e.g. I am an extraordinary person). Participants rated how much they agreed with each item ranging from 1 (strongly disagree) to 5 (strongly agree) and higher scores indicated greater disposition for that variable. Reliability estimates have been shown to range between .49-.76 in previous studies [50].

#### Familiarity and knowledge of computers and phishing

The Familiarity and Knowledge of Computers and Phishing consisted of two parts. Firstly, participants were asked to provide a definition of phishing. This has been used in studies such as [10] and [51] to examine phishing-based perceptions and understanding. The definitions were analysed and coded independently by three research assistants based on the accuracy of the definition from 0–3, with a higher score indicating greater accuracy. For example, a definition which scored 0 is “An email where it can be interpreted in a number of different ways due to how it is said” or “no clue” whilst a perfect scored definition is “Emails with which the sender/s have the intention to reveal your personal information (credit card details, passwords to security accounts, etc).” Average inter-correlation between raters was .80 and further definition examples can be found in Table A in S1 Appendix. After the participants had attempted to define phishing, an accurate definition was provided to them.

Secondly, to capture pre-test self-reported competence in phishing detection, participants indicated how much they agreed that they could detect phishing email (using 5-responses ranging from Strongly Disagree to Strongly Agree) (similar to [29]).

#### Risk profile questionnaire (RPQ; [52])

The seven-item questionnaire measures different behavioural tendencies for personal and online security. It is employed as a measure of self-reported knowledge of computers, with each question being treated as a separate variable. Participants answer Yes or No to each of the questions like “Have you ever noticed the "padlock" icon that appears in the lower right portion of your browser for certain websites?” and “Have you ever stopped a transaction or avoided a transaction because you did not see a seal of approval such as Verisign listed at checkout?”. As these items were analysed separately, they are listed in Table B in in S1 Appendix.

#### Demographics

Age, gender, education, country of origin, Australian residency prior to university, fluency in English, years lived in Australia and having English as a first language were collected.

### Procedure

Participants were provided with a weblink for the experiment and they completed it in their own time. The order of the measures was Demographics, Esoteric Analogies Test (EAT), Personality Inventory, Familiarity and Knowledge of Computers and Phishing, and Risk Profile Questionnaire (RPQ), and the Phishing Detection Task (PDT). Stimuli in the phishing detection task were presented in a randomised order for each participant to remove order effects. The Phishing Detection Task was given last to make sure that the responses on this test would not bias the responses on the other questionnaires. Participants also completed a feedback questionnaire afterwards, though these results are not within the scope of this paper.

## Results

### Descriptive statistics and reliability estimates

The descriptive statistics and reliability estimates for all measures are shown in Table 3.

**Table 3:** Descriptive statistics and reliability estimates of other variables.

	Mean (%)	SD	Min	Max	Cronbach’s α
***Phishing Detection Task (Phishing Email)*** Email Detection Accuracy	73.9	19.1	20.0	100.0	.80
Behavioural Response Confidence	74.6	15.3	17.0	100.0	.93
Perceived Email Maliciousness	66.4	14.4	24.9	100.0	.86
***Phishing Detection Task (Genuine Email)***					
Email Detection Accuracy	78.1	18.7	15.0	100.0	.81
Behavioural Response Confidence	74.0	15.9	23.0	100.0	.94
Perceived Email Maliciousness	27.8	15.7	0.0	70.4	.89
***Personality***					
Agreeableness	3.4	.7	1.5	5.0	.60
Conscientiousness	2.9	.7	1.5	4.3	.57
Extraversion	3.6	.6	2.0	5.0	.51
Originality/ Intellect	3.2	.6	1.5	4.8	.44
Honesty/ Propriety	3.2	.6	1.4	4.6	.45
Resilience	2.9	.7	1.3	4.5	.61
***EAT***					
EAT accuracy	69.4	15.5	20.8	95.8	.72
EAT confidence	75.8	11.8	43.1	97.8	.88
***Risk Profile Questionnaire*** Padlock Icon Attention	57.3	49.6	0.0	100.0	-
Destroying Old Documents	56.7	49.7	0.0	100.0	-
Valuable Possession Protection	68.0	46.8	0.0	100.0	-
Computer Update Installation	51.3	50.1	0.0	100.0	-
Seek Online Retailer Legitimacy	50.0	50.2	0.0	100.0	-
Website Privacy Policy	36.7	48.4	0.0	100.0	-
Online Checkout Seal of Approval	38.0	48.7	0.0	100.0	-

Note: “-”indicates that internal consistency reliability was not calculated due to use of only one item

**Fig 3:**
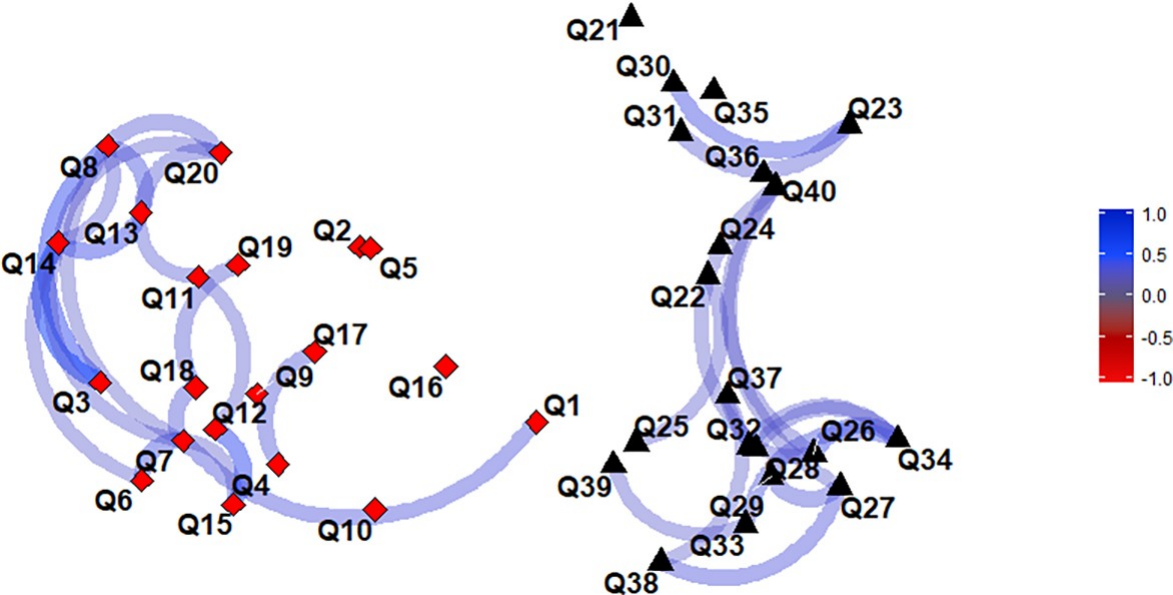
Network plot of correlations between all 40 email items. Q1-20 are phishing email while Q21-40 are genuine email. Correlations stronger than |.3| are shown. Red diamonds represent phishing email whilst black triangles represent genuine email.

Phishing detection accuracy was 73.9%. What this means is that overall, participants kept 26.1% of the malicious email. Similarly, email detection accuracy for genuine email was 78.1%, leaving 21.9% of genuine email trashed. Perceived email maliciousness was 66.4 for phishing and 27.8 for genuine email, indicating that overall participants were able to differentiate between these different types of email. Cronbach’s alpha for accuracy in the Phishing Detection Task was .80 for phishing email and .81 for genuine email; and .86 and .89 for perceived email maliciousness of phishing and genuine email respectively, pointing to a high internal consistency with which people approached phishing and genuine email. It should be noted, however, that these statistics do not provide information about the internal structure of the test [53], an issue which is outside of the scope of this paper. Some information about it, however, is contained in the plot of correlations reported below (Fig 3). For instance, the fact that there are two distinct clusters of correlations in the responses for phishing and genuine email respectively suggests that there are at least two distinct accuracy factors/clusters, one for each type of email. That is, participants’ accuracy varied systematically within each email type as there is a robust ‘connectivity’ between responses within each cluster, but not across the different types of email.

Statistics for confidence, intelligence and personality measures are within the expected range and similar to those reported in previous studies [20, 50].

### Phishing susceptibility and false positives

For phishing email, on average, participants responded by trashing the email 66%, keeping the email 26%, and seeking more information 8% of the time. For genuine email items, the corresponding averages were 22%, 71%, and 7% respectively. The distribution of how often participants chose each behavioural response to phishing and genuine email are shown in Fig A in the S1 Appendix.

A significant negative correlation was found between phishing and genuine email detection accuracy, *r* = -.29, *p <* .*01)*. A network plot of the correlations in accuracy of responses between email items is shown below in Fig 3. The plot indicates that there are two robust yet distinct clusters of correlations, one for responding to phishing email and the other for genuine email. Thus, the plot indicates that there is a strong separation of accuracy tendencies for phishing and genuine email.

Correlations between phishing susceptibility and individual differences variables captured in this study are summarised in Table 4.

**Table 4:** Correlations between phishing susceptibility and individual difference measures.

	Email Detection Accuracy		Perceived Maliciousness	
	Phishing	Genuine	Phishing	Genuine
***In-task Variables***				
Perceived Maliciousness *(Phishing Items)*	.67^**^	-.12	-	.27^**^
Confidence (*Phishing Items)*	.24^**^	.09	.33^**^	-.27^**^
Perceived Maliciousness *(Genuine Items)*	.07	-.61^**^	.27^**^	-
Confidence *(Genuine Items)*	-.01	.23^**^	.03	-.52^**^
***Individual Difference Variables***				
Esoteric Analogies Test Accuracy	.28^**^	.24^**^	.18^*^	-.17^*^
Esoteric Analogies Test Confidence	.10	.08	.12	-.12
Accuracy of Phishing Definition	.32^**^	.14	.29^**^	-.19^*^
Self-Reported Competence in Detecting Phishing *(Prior to PDT)*	.16^*^	.08	.28^**^	-.13
***Personality***				
Honesty/ Propriety	.10	.02	.03	.05
Extraversion	-.04	.01	-.06	-.03
Resilience	.03	-.02	.09	.09
Agreeableness	.02	.04	.06	-.01
Conscientiousness	.06	-.04	.01	.09
Originality/ Intellect	.07	.10	-.00	-.12
***Demographics***				
Age	-.01	-.02	-.02	.04

* p< .05

** p<. 0.01

Perceived maliciousness significantly and strongly correlated with email detection accuracy, with a positive correlation for phishing detection (.67, *p* <. 01) and a negative correlation for genuine detection (-.61, *p* <. 01). A small, yet significant positive correlation was found between perceived maliciousness of phishing and genuine email (.27, *p* <. 01). Email detection confidence shared a significant, albeit small positive correlation with email detection accuracy for both phishing and genuine email items (.24 and .23 respectively, *p* <. 01). Phishing email detection confidence significantly correlated moderately and positively with perceived maliciousness in phishing email and weakly and negatively with perceived maliciousness in genuine email (.33 and -.27, *p* <. 01). Genuine email detection confidence correlated significantly with perceived maliciousness in genuine email, with a strong and negative correlation (-.52, *p* <. 01). EAT accuracy score shared a significant, albeit small, positive correlations with both phishing and genuine accuracy of detection and perceived maliciousness in phishing (.28, .24, *p* <. 01 and .18, *p* <. 05 respectively). EAT accuracy score also shared a small and negative correlation with perceived maliciousness in genuine email (-.17, *p* <. 05). Phishing definition accuracy shares significant moderate positive correlations with accuracy of phishing detection and perceived maliciousness of phishing email (.32 and .29, *p* <. 01 respectively). There was also a small and negative significant correlation between phishing definition accuracy and perceived maliciousness in genuine email (-.19, *p* <. 01). Self-reported competence in detecting phishing (prior to the PDT) correlated significantly, but weakly with both detection accuracy and moderately with perceived maliciousness of phishing email (.16, *p* <. 05 and .28, *p* <. 01 respectively). Personality variables shared no significant and meaningful correlations with any of the variables of interest. Thus, they were excluded from any further analyses.

Furthermore, individuals with English as a first language detected genuine email more accurately (M = 80.0%, SD = 18.7) and perceived genuine email as malicious less (M = 33.4%, SD = 17.3) than individuals without English as a first language, (M = 72.9%, SD = 17.8), *t*(146) = -2.03, *p* = .044, and (M = 25.9%, SD = 14.7), *t*(146) = 2.61, *p* = .010. Further post-hoc contrast analyses with Scheffe adjustments were conducted for those individuals who did not have English as a first language based on their frequency of use of an English dictionary. These participants were re-categorized into three groups of English dictionary use (Never, Rarely-Sometimes, Often-Always). Contrasts could not compare Never as there were only three individuals within the group, and no significant differences in phishing susceptibility were found between the other two groups. Lastly, individuals who had noticed the padlock icons on websites were able to detect phishing email more accurately (M = 76.7%, SD = 18.6) than those who did not notice the icons (M = 70.0%, SD = 19.2), *t*(146) = -2.16, *p* = .033. No other significant relationships were found between other variables and email detection accuracy.

Gender and age did not share any significant relationships with the phishing susceptibility variables. All t-test outcomes can be found in Table C in S1 Appendix.

**Fig 4:**
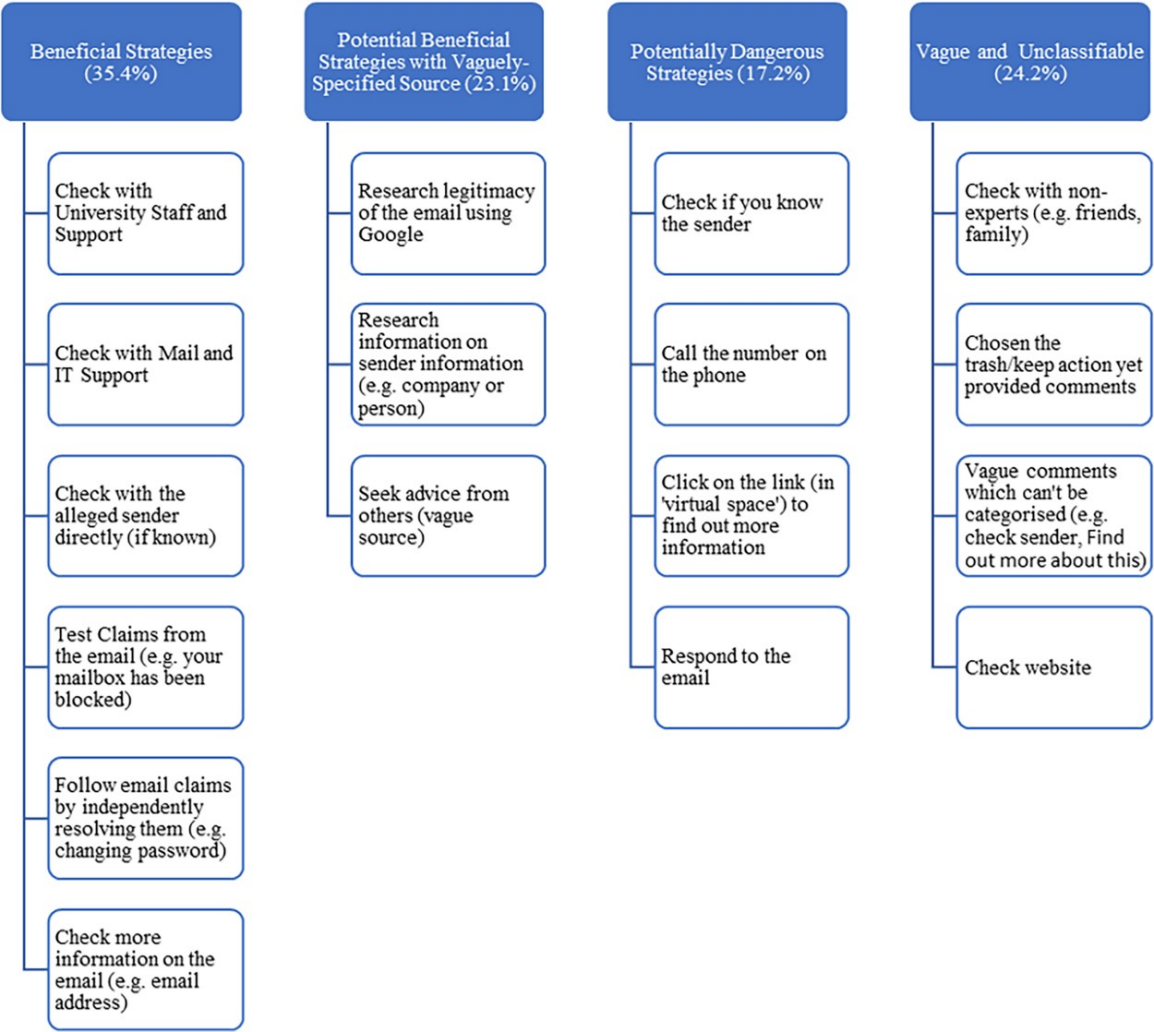
List of information sought after by participants when seeking more information on the PDT.

Seeking more information was an option that participants rarely used—the maximum percentage seeking more information being 20.3% indicated for a spear phishing email from “a University of Sydney IT administrator”, asking participants to update their email account. All other email had information seeking behaviour ranging only between 1.4 to 14.2%. Extending our investigation into how participants sought more information, we categorised their responses into four several broader responses (as shown in Fig 4).

Participants were most likely to use beneficial ‘seeking more information’ strategies via legitimate verification sources, including seeking advice from IT personnel, university staff and directly contacting the sender outside of the email if they knew them (35.4%). The second most common behaviours were vaguely specified and thus difficult to classify in terms of whether they were beneficial or potentially dangerous (24.2%). The examples include conduct a search, “contact someone else/ human about it”, unsubscribe, ignore it, consider the relevance, ask friends/family/peers about it, check credibility of the source/sender (not specifying how). Checking on the website domain also counted as vague as, depending on the approach, it could be both a beneficial or dangerous strategy (the site itself could be phishing). Participants also sought out what were classified as Potential Beneficial Strategies, but with vaguely-specified sources (23.1%). The examples include “google it”, research it/company/source/credential/sender/what to do in the situation, ‘secure email’, check the link. The final (and the least common category) was classified as Potentially Dangerous Strategies (17.2%). The examples include: to reply to email/call the sender, click on a link, make further contact, open/read the attachment, reply with a joke (asking whether they know how to spell, or to provide their details, or to suggest to the sender to learn basic English).

We also investigated the correlations between frequency of seeking more information and other measured variables. Frequency of seeking more information was weakly and positively correlated with the phishing definition mean score and self-reported confidence in handling phishing (.27, p = .001 and .20, p = .016 respectively). It also moderately and positively correlated with EAT accuracy (.34, p < .001) and Phishing Email Detection Accuracy (.37, p < .001). Finally, participants who indicated that they noticed the padlock icon on websites were significantly more likely to seek more information (M = .09, SD = .10) than those who did not (M = .06, SD = .07), p = .021.

### Regression analyses

To address the hypotheses, several sequential regression analyses were conducted. To address the hypotheses relevant to the first aim, two analyses were performed for a) Phishing Detection Accuracy and b) Perceived Maliciousness of Phishing Email. Similarly, to address Aim 2, two analyses were performed for a) Genuine Detection Accuracy and b) Perceived Maliciousness of Genuine Email. Thus, overall four regression analyses were completed to address the hypotheses, with dependent variables being phishing and genuine email maliciousness perception and phishing and genuine email detection accuracy.

For the two models examining Phishing and Genuine Detection Accuracy, we used the following steps. For Step 1 of the model, control variables of age, gender and English as a first language were entered. Step 2 included Esoteric Analogies Test Accuracy and Confidence scores, and Step 3 included “phishing awareness/competence” measures: capacity in handling phishing email, PDT phishing/genuine email confidence and awareness of padlock icons. In Step 4, Perceived Maliciousness of the relevant email type (i.e. phishing or genuine) was included. The rationale behind this order was based on two important considerations. Firstly, time precedence/stability, thus age, gender and English as a first language were entered as block 1, intelligence and associated confidence as block 2. Secondly, we decided to have ‘phishing awareness’ variables in block 3 and Perception of Maliciousness of the relevant email in block 4 to investigate whether there might be a mediation effect (full or partial) of Perceived Maliciousness in the predictions that ‘phishing awareness’ variables had on phishing and genuine email detection accuracy. That is, if the predictive power of the ‘phishing awareness’ variables lessen or disappear after the introduction of the perceived maliciousness variable, it would signal that this prediction was mediated partly or fully, respectively, by the perceived maliciousness variable (see [54] for a review).

In two separate regression models, we regressed Perceived Maliciousness of Phishing Email and Perceived Maliciousness of Genuine Email on the same 3 blocks specified above to examine what predicts these important perceptions and to unpack the nature of the possible mediation (see [54] for a review).

In addition to reporting the percentage of variance incrementally predicted by each block, we calculated unique variance estimates (squared values of semi-partial correlations) to highlight the effect sizes for each variable in the model [54, 55], and we discussed them in relation to the relevant hypotheses.

Correlations between all predictor variables within the regression models are shown in Table 5 and all steps for the regression models are summarised in Table 6.

**Table 5:** Correlations between variables used in regression models.

	2	3	4	5	6	7	8	9	10	11	12	13	14
1. PDT Phishing Email Detection Accuracy	-.27**	.68**	.07	.02	-.10	.16	.27**	.01	.32**	.16*	.18*	.24**	-.01
2. PDT Genuine Email Detection Accuracy	1	-.12	-.61**	-.00	-.06	.17*	.24**	.08	.14	.08	-.03	.09	.23**
3. PDT Phishing Maliciousness Perception		1	.28**	.01	.13	.15	.18*	.12	.29**	.28**	.14	.32**	.03
4. PDT Genuine Maliciousness Perception			1	.03	.06	-.21*	-.17*	-.13	-.19*	-.11	-.05	-.26**	-.51**
5. Age				1	.13	-.10	.00	-.01	-.01	.06	.02	.09	.02
6. Gender					1	-.02	-.04	.06	.04	.17*	.15	.23**	.20*
7. English as a First Language						1	.10	-.11	.14	.06	.20*	.15	.08
8. EAT Accuracy							1	.46**	.35**	.23**	-.01	.20*	.21*
9. EAT Confidence								1	.25**	.17*	.06	.30**	.37**
10. Phishing Definition Score									1	.34**	.23**	.14	.18*
11. Perceived Capacity in Handling Phishing										1	.15	.35**	.31**
12. Awareness of Padlock Icon											1	.13	.10
13. PDT Phishing Confidence												1	.80**
14. PDT Genuine Confidence													1

p < .05*, p < .01**, p < .001***

**Table 6:** Results of sequential regression models predicting phishing and genuine maliciousness perception and detection accuracy.

		Phishing Detection Accuracy						Maliciousness Perception					
	Predictor	Phishing Email			Genuine Email			Phishing Email			Genuine Email		
		ΔR^2^	β	Unique Variance	ΔR^2^	β	Unique Variance	ΔR^2^	β	Unique Variance	ΔR^2^	β	Unique Variance
Step 1		.04			.03			.04			.05		
	Age		.05	.23%		.02	.03%		.01	.01%		.00	.00%
	Gender		-.11	1.10%		-.07	.42%		.13	1.56%		.06	.41%
	English as a First Language		.16	2.53%		.17*	**2.77%**		.15	2.32%		-.21*	**4.24%**
Step 2													
		.06**			.05*			.03			.03		
	Age		.05	.22%		.02	.03%		.01	.01%		.00	.00%
	Gender		-.09	.81%		-.05	.27%		.13	1.63%		.07	.43%
	English as a First Language		.13	1.59%		.14	1.85%		.14	1.93%		-.21*	**4.09%**
	EAT Accuracy		.26**	**4.84%**		.23*	**3.82%**		.14	1.56%		-.09	.65%
	EAT Confidence		.00	.00%		-.01	.00%		.06	.29%		-.11	.90%
Step 3													
		.11**			.05			.12***			.25***		
	Age		.02	.05%		.02	.03%		-.02	.05%		.00	.00%
	Gender		-.17*	**2.50%**		-.08	.59%		.04	.16%		.16*	**2.20%**
	English as a First Language		.04	.17%		.12	1.33%		.07	.41%		-.14	1.70%
	EAT Accuracy		.18*	**2.27%**		.20*	**2.74%**		.05	.17%		-.05	.20%
	EAT Confidence		-.10	.64%		-.09	.49%		-.04	.10%		.09	.60%
	Phishing Definition Score		.22*	**3.81%**		.06	.30%		.20*	**2.93%**		-.11	1.00%
	Perceived Capacity in Handling Phishing		-.01	.01%		-.03	.08%		.11	.86%		.07	.40%
	Awareness of Padlock Icon		.12	1.29%		-.07	.43%		.03	.07%		.02	.00%
	PDT Phishing Confidence		.21*	**3.44%**		-	-		.25**	**4.56%**		-	-
	PDT Genuine Confidence		-	-		.23*	**3.97%**		-	-		-.56***	**23.90%**
Step 4													
		.33***			.28***								
	Age		.04	.12%		.02	.04%		-	-		-	-
	Gender		-.19*	**3.36%**		.02	.04%		-	-		-	-
	English as a First Language		.00	.00%		.03	.09%		-	-		-	-
	EAT Accuracy		.15*	**1.53%**		.17*	**1.89%**		-	-		-	-
	EAT Confidence		-.07	.35%		-.03	.05%		-	-		-	-
	Phishing Definition Score		.10	.70%		-.01	.01%		-	-		-	-
	Perceived Capacity in Handling Phishing		-.08	.44%		.01	.02%		-	-		-	-
	Awareness of Padlock Icon		.10	.93%		-.06	.29%		-	-		-	-
	PDT Phishing Confidence		.06	.22%		-	-		-	-		-	-
	PDT Genuine Confidence		-	-		-0.13	1.03%		-	-		-	-
	PDT Phishing Maliciousness Perception		.64***	**33.28%**		-	-		-	-		-	-
	PDT Genuine Maliciousness Perception		-	-		-.65***	**28.29%**		-	-		-	-
Overall % of Variance Accounted for by Model		53.90%			41.00%	** **	** **	19.30%			33.20%		

*p <* .05*, *p <* .01**, *p <* .001*** significant predictors are in bold.

#### Phishing susceptibility

Block 1 predicted between 3 and 5% of variance in both dependent variables. Both predictions were not significant (p > .05), these results were consistent with a pattern of relationships between gender, age and English as a first language with the dependent variable. The addition of the second block (EAT accuracy and confidence), predicted an additional 6% (p < .05) in phishing email detection accuracy with only intelligence (EAT accuracy) being a significant predictor in this block as well as subsequent blocks. Thus, supporting hypothesis 1a, intelligence (EAT accuracy) positively predicted phishing email detection accuracy contributing uniquely almost 5% of variance to the prediction in block 2, and 1.5% in block 4 uniquely above and beyond all other variables in the model. This relationship was not statistically significant in the model predicting the perception of maliciousness, thus hypothesis 1b was not supported.

The addition of the ‘phishing awareness’ variables in the 3rd block to the sequential regression predicted an additional 11% of the variance in phishing email detection accuracy (p < .01) and 12% in predicting perceived maliciousness of phishing email (p < .01). Phishing Definition Score (unique variance = 3.81% and 2.93% respectively) and Phishing Confidence (unique variance = 3.44% and 4.56%) variables were the only significant predictors in this block for both dependent variables. Thus, there was a partial support for hypothesis 2a and 2b with PDT phishing confidence positively predicting both phishing email detection accuracy and maliciousness perception. Subjective measures of knowledge of phishing and computers (reported competence in phishing detection and awareness of padlock icon), however, were not statistically significant predictors in any of the models, thus providing only partial support for hypotheses 3a and b.

The addition of phishing email maliciousness perception in block 4 offered an additional and substantial 33% change in the variance accounted for (p < .001) in the overall prediction of accuracy of detection of phishing email, over and above of all other variables in the regression. Thus, hypothesis 4 was fully supported.

Notably, the addition of this important variable changed the sizes and, in many cases, the significance of the other predictors in the model. In particular, intelligence (EAT accuracy) positively predicted phishing email detection accuracy contributing uniquely almost 5% of variance to prediction in block 2, but in model 4, this amount dropped to 1.5%. Phishing Definition Score and Phishing Confidence both lost statistical significance as predictors in block 4. Hence, perceived maliciousness partly (for EAT accuracy) and fully mediated predictions of Phishing Definition Score and Phishing Confidence variables on Phishing detection accuracy. Both variables, however, were statistically significant predictors of maliciousness perception with each variable predicting 2.93% and 4.56% of the variance uniquely. Therefore, there was support for hypothesis 3b, with phishing definition score positively predicting maliciousness perception but not phishing email detection accuracy.

The phishing definition score and PDT phishing confidence were significant predictors of phishing email detection accuracy in the regression model that did not contain maliciousness perceptions. When maliciousness perception was entered, these predictions become non-significant. These variables, however, were statistically significant predictors of maliciousness perception, thus suggesting the presence of an indirect effect on phishing detection accuracy via their relationships with the maliciousness perception.

#### False positives

A similar pattern of results was revealed for genuine email detection accuracy. Block 1 with control variables did not predict any significant amount of variance in both detection accuracy and maliciousness perception in genuine email. English as a first language, however, was a significant predictor in this block for both models, predicting 2.77% and 4.24% of variance in accuracy of detection and perception variables respectively. This significant effect disappears in the subsequent blocks 3 and 4, and thus is omitted in further discussions.

The addition of the second block (EAT accuracy and confidence) predicted 5% (p < .05) in phishing email detection accuracy, with only intelligence (EAT accuracy) being a significant positive predictor in this and subsequent blocks, thus, supporting hypothesis 5a. As with phishing email, EAT accuracy’s percentage of unique variance declined from block 3 to block 4, with the unique variance predicted above and beyond other variables in the model dropping from 3.82% to almost 1.89%. This relationship was not statistically significant in the model which predicted the perceived maliciousness in genuine email. Thus, hypothesis 5b was not supported.

The addition of the third block with the “phishing awareness” variables did not introduce a significant amount of additional variance in prediction of the accuracy detection (R squared change = .046, p = .13). However, PDT genuine item confidence positively predicted the detection with 3.97% of unique variance. This significant prediction disappears in block 4 after entering perceived maliciousness in genuine email in the model.

When perceived maliciousness of genuine email was regressed on relevant variables, the ‘phishing awareness’ variables as a block contributes a substantial 25% of additional variance (p < .001), with PDT genuine email confidence negatively predicting maliciousness perception of genuine email (unique variance = 23.90%). This supports hypothesis 6b.

Hypotheses 7a and b were not supported as neither objective nor subjective measures of knowledge of phishing/computers were significant predictors of detection accuracy or perceived maliciousness of genuine email. Finally, hypothesis 8 was strongly supported, with maliciousness perception in genuine email negatively predicting genuine email detection accuracy as entered in block 4, adding an additional 28.29% (p < .001) unique variance to the prediction. Once again, it should be noted that PDT genuine confidence was a significant predictor of genuine email detection accuracy in Step 3 of the regression model, becoming non-significant in Step 4 when genuine email maliciousness perception was entered.

#### Gender differences

Surprisingly, there was mixed support for hypothesis 8, with gender not being a significant predictor as entered in the first block, but becoming a significant predictor in some subsequent blocks. Given that relevant correlations were weak and non-significant, the subsequent ‘gaining’ of statistical significance is likely to be a sporadic result (see [55] and [56] for reviews).

#### Personality variables

Adding personality variables in any of the regression equations did not contribute any significant amount of additional variance. This was consistent with the correlations between personality variables and the variables of interest. Thus, supporting hypotheses 10a and b, personality shared negligible relationships with (a) detection accuracy of phishing and genuine email and (b) perception of maliciousness of phishing email.

### Characteristics of the most successful phishing email

The characteristics for the four most successful phishing email items and four most false-positive inducing genuine email items are shown in Table 7. The most successful phishing email and the most false-positive inducing genuine email can be found in S1 Appendix as Figs B and C.

**Table 7:** Characteristics of most successful phishing email and least successful genuine email.

	Phishing				Genuine			
Item Number	17	1	3	10	34	25	28	32
Email Word Length	1075	181	67	51	223	84	15	254
Target Group	LT	SP	LT	LT	SP	SP	G	LT
Contact Method	H/ER	H	H	H	H	ER	H	ER
Number of URLs	6	7	1	1	4	1	1	7
***Suspicion-Inducing Characteristics***								
Misspellings and Grammatical Errors	Yes	No	Yes	Yes	Yes	No	No	Yes
Vague Recipient	Yes	Yes	Yes	No	Yes	Yes	No	Yes
Pressure	Yes	Yes	No	Yes	Yes	No	No	No
Suspicious Email Domain	Yes	No	No	No	No	No	No	No
Suspicious URL	Yes	Yes	Yes	No	Yes	No	Yes	Yes
Asks for Confidential Information	No	No	No	No	No	No	No	No
***Trust-Inducing Characteristics***								
Official Email Domain	No	No	No	No	No	Yes	No	Yes
Official URL	No	No	No	No	No	No	No	Yes
Use of URLs with HTTPS	Yes	No	No	No	Yes	No	No	No
Number of Suspicion-Inducing Characteristics	5	3	3	2	4	1	1	3
Number of Trust-Inducing Characteristics	1	0	0	0	1	1	0	2
Behavioural Percentages on Email								
Kept	.54	.47	.46	.44	.63	.60	.54	.54
Trashed	.38	.48	.44	.45	.30	.32	.38	.41
Sought Further Information	.07	.05	.09	.11	.07	.09	.07	.05

SP = Spear Phishing, LT = Loosely-Targeted, G = Generic; H = Hyperlink, ER = Email Reply

The most trust-inducing phishing email generally had more specific target victims, with three being loosely-targeted and one spear-phishing. Surprisingly, three of these email items did not contain any trust-inducing characteristics with the other email only containing one trust-inducing characteristic of an HTTPS URL. These email items also varied in the number of suspicion-inducing characteristics, ranging from 2 to 5 such characteristics, and every suspicious email characteristic was found in these top four difficult email items.

Within the four least kept, but genuine email, no particular target group or contact method appeared to be commonly found in these email items. Furthermore, these items did not have particularly more suspicious-inducing characteristics than other email, although two of the genuine email messages had a high number of such characteristics.

## Discussion

The overarching goal of this study was to determine factors that predict phishing susceptibility, focusing on both personal and stimuli characteristics. While focusing on phishing susceptibility, we also collected information on how people responded to genuine email (false positives). Thus, this study’s aims were three-fold: 1) to determine the relationships between phishing susceptibility and different individual difference variables, 2) to determine the relationships between the frequency of false positives and different individual difference variables and 3) to determine the email characteristics which induced the greatest likelihood of phishing success and false positives. To achieve these goals and to mitigate the problems associated with previous research, we developed and used a novel 40-item phishing detection task with stimuli taken from current real-life email targeting the general public and higher education students. To deepen our understanding of cognitive and behavioural processes involved in phishing detection and frequency of false positives, we also employed a novel decision-making process using perception of maliciousness and behavioural actions on email to characterise email instead of a simple phishing/ non-phishing taxonomy. The maliciousness perception of, and behavioural judgements for email were then used as indications of phishing susceptibility and false positives.

The current study employed measures of intelligence, Big 6 personality (with an additional Honesty trait compared with Big 5 personality), and both on-task and other-task confidence. Following previous research, objective and subjective measures of computer and phishing knowledge, gender and English fluency were also examined.

### The phishing detection task (PDT)

We aimed to examine the relationships between online deception detection and different individual difference variables. However, there is currently no standardised measure for phishing detection. Thus, we created the PDT with participants judging perceived maliciousness, action on and confidence about the action for a variety of phishing and genuine emails. We developed this test to ensure that we captured internally consistent tendencies of phishing susceptibility and false positives relevant to our population of interest: university students and academia in general. Although novel, the PDT has good internal consistency estimates for phishing and for genuine email (Cronbach’s α were .80 and .81 respectively). Although not informing us about the internal structure of this novel test, and providing a lower boundary of reliability [53], these high alpha reliability estimates for phishing and for genuine email suggest that people provided consistent responses on the PDT. This was also the case for confidence and perceived maliciousness variables (Cronbach’s α ranged between .86 - .94). This extends previous research where limited numbers (often just one) of phishing and genuine email were used and attests to the fact that people respond to both sets of email with robust consistency.

It also became apparent that people responded differently to phishing and to genuine emails based on the network plot of correlations between email items. Thus, only having a phishing/non-phishing dichotomy appears to be too simple, and further research into addressing the role of false positives would be beneficial.

### Phishing susceptibility and false positives

Several key findings emerged. Firstly, people are capable of detecting phishing email within an experimental setting. On average, three in four email items were detected accurately for both phishing and genuine email, with some individuals even having full scores for email detection accuracy. This high accuracy rate is similar to scores from Pattinson et al. [22]’s informed participants, and may be attributed to participant awareness of the task requiring them to discriminate phishing from genuine email. Although phishing relies on the unawareness of victims, the high accuracy rates for informed participants suggest that individuals do have the capacity to accurately distinguish phishing and genuine email. This is a positive finding, as in the real world, it is clear that a failure to detect an actual attack can have very serious consequences.

Secondly, despite the relative high accuracy in detecting phishing, there are also general weaknesses in behaviour in responding to potential phishing, namely, the hesitation to seek more information. That is, participants responded with seeking more information only 8% of the time for phishing email and only 7% for genuine email. Although seeking more information may delay task performance in real world contexts, it is often crucial to avoiding falling for phishing. Closer examination of participants’ information seeking behaviour comments shows that although most of the information seeking behaviours are beneficial and safe (e.g. personally checking information online, seeking advice from others), a substantial number of responses involved contact and opening links/attachments which may result in a greater likelihood of being phished. Further increasing this risk, many people appear to be naïve about the risks of phishing email, with self-reported competence in detecting phishing not predicting actual phishing susceptibility nor the frequency of false positives. Thus, the combination of exaggerated self-beliefs in competence, reluctance to seek further information and/or seeking information in a potentially dangerous fashion could lead to increases in phishing success. This finding highlights the potential value of training in increasing both awareness and competence.

Thirdly, our regression models were able to successfully account for 53.9% and 41% of variance in phishing susceptibility and false positives susceptibility using psychological variables as predictors overall. We were able to successfully replicate and extend the current literature with our finding that individuals who are more intelligent (with a mixed measure of Fluid and Crystalized intelligence) are more capable of discriminating between phishing and genuine email [6, 28]. The most potent predictor, however, in both models was perception of maliciousness of phishing and genuine email, which as a single variable predicted a massive unique percentage of variance in both models (33% and 28% respectively). In addition to being a key direct predictor of the accuracy of detection of both types of email, this variable mediated the predictions of several ‘phishing awareness” variables—Phishing Definition Score (for both phishing and genuine email) and on-task PTD confidence (for phishing email). Overall, these results suggest that it is important to capture these perceptions in order to understand the nature of phishing susceptibility and false positives. Extending the results of previous research, people who were more knowledgeable and confident in dealing with phishing were more capable of detecting phishing and genuine email, but this prediction was indirect, via the perceptions of maliciousness of the relevant email [10, 26]. This finding is easy to overlook if the maliciousness perception variable is not captured, thus contributing to inconsistent results between the studies.

Finally, among the ‘phishing awareness’ variables, it was the objective indicators—phishing definition score provided by participants and on-task confidence in behavioural actions on email—that predicted perception of maliciousness. Self-report measures hold little predictive power for either accuracy of detection or perception of maliciousness for both types of email above and beyond other variables. This is consistent with more general findings in psychology that more objective metrics share more meaningful relationships with actual behaviour, decision-making and performance [28].

Although no causal interpretations can be drawn from this study, overall, these and previous findings [e.g. 29, 33, 57] provide support for the need for training about phishing aiming to reduce phishing susceptibility. In this regard, Kumaraguru and colleagues [57] state that “while automated detection systems should be used as the first line of defense against phishing attacks, user training offers a complementary approach to help people better recognize fraudulent emails and websites”. (p. 7:1). Similar to most employees and executives in various corporate and public companies, student populations across the world should receive targeted and timely training in phishing. Such education measures should aim to promote information-seeking behaviour when the individual is suspicious of phishing, and increase awareness of not only the existence of phishing but also the email characteristics which are most successful. Furthermore, as those with more knowledge about computers tend to have lower phishing susceptibility and false positives, we strongly recommend these training measures be particularly targeted to populations with weaker computer backgrounds.

### Limitations and future direction

As there is no standardised measure for phishing detection, the current study designed and used a novel phishing detection task. This means that our results are not strictly comparable to literature based on various other sets of email for the phishing detection task. Although all variables measured within the task had good internal consistency, further studies should use the task to replicate these findings. We note that devising good sets of email for such studies is challenging, all the more so as they should make use of the newest and most current phishing items to maintain the task fidelity. Therefore, we suggest that future studies source a range of the most recent phishing email targeting an audience of interest to update the Phishing Detection Test used in this study. We also recommend that instead of employing a simple ‘phishing/non-phishing’ taxonomy, future studies follow our work and also capture perceptions of maliciousness and ecologically valid behavioural actions on email (the latter may be different in different populations).

Although we examined a large variety of both novel and previously examined variables within the paradigm, we did not measure and control for some variables which have been shown to relate strongly with phishing detection, namely, attention to stimuli as well as baseline trust [13,15]. Within experimental settings, participants may have attended to email more vigilantly and achieved higher accuracy scores as greater attention has been shown to relate with higher phishing detection rates [15]. However, we believe that these inflated scores are valid measures of phishing detection as they point to an individual’s maximal capacity for discriminating email. Nevertheless, future studies should also include attention and trust measures. There were also limitations with our Risk Profile Questionnaire which asks participants whether they had noticed the padlock icon “in the lower right portion of the browser”. Modern browsers often have the padlock icon in the browser address bar. The Risk Profile Questionnaire had been previously used in other publications and thus we did not make any alterations for consistency reasons [52]. However, we acknowledge that future studies would benefit from adapting the items to match current contexts more accurately.

A further limitation is the use of a phishing detection task where participants knowingly discriminated between phishing and genuine email. When a real-life phishing attack occurs, people remain uninformed and may also be subject to various contextual factors, such as time pressure. Studies, however, have shown individuals to be less accurate when discriminating phishing when uninformed than informed [22]. Although an informed participant setting allowed us to more easily examine a large number and variety of email, we strongly suggest future studies replicate the current study with uninformed participants. Additionally, in authentic settings, the proportion of phishing email is very small, compared with genuine email. So replications should also account for this.

This expectancy bias also has implications for predictive validity, whether participants who are good phishing detectors in-lab are also good in real-world settings. Recently, Canfield et al. [58] examined the relationships between a behavioural phishing detection measure and real-life computer security outcomes from the Security Behavior Observatory (SBO), a client program which monitors and collects long-term computer behavioural data from users, finding no significant relationships. Aligning with Canfield et al.’s conclusions, we believe that such empirical research is able to capture phishing detection performance. However, the complexity of real-world contexts makes it difficult to link such performance to real-world outcomes. In particular, participants are primed to detect phishing email during experiments whereas they are completely uninformed in real-world contexts. We believe that participants can demonstrate their best level of performance for phishing detection in experimental contexts, and the real-world contexts holds multiple factors which could diminish their capacity including time constraints, greater online exposure, and reliance on malware detection software. Although challenging, further investigation into objective real-world outcomes with in-lab phishing detection performance would be beneficial for determining predictive validity.

Lastly, contrasting prior research, our results found mixed gender differences in the final blocks of the regression model, such that women were predicted to judge genuine email as more malicious but also to be better at distinguishing phishing email [32, 37]. However, gender was not a significant predictor in prior blocks of the regression models, and had weak and non-significant relationships with measures of phishing and false positive susceptibility. Thus, it is likely that this result was sporadic and may have been due to the different sample population used (first year psychology students). As such, further replication of our study with other populations would be beneficial.

Our work is complimentary to the current best practices for protecting users from phishing attacks. These involve a combination of technological approaches and user training. The most recent academic literature, such as Wash et al. [59] and Siadati et al. [60], highlight the continuing need for phishing training as opposed to purely technical approaches, particularly recommending embedded training as the current best practice. Essentially, this delivers training through the user’s day-to-day interactions where they click on links in their own inbox and are subsequently asked to assess the safety of that email and the clicked link. They are then presented with feedback. Such systems can deliver email purely for training or can operate on real email intended for the user with the system itself needing to distinguish safe links. Following these recommendations, there are several commercial vendors for such embedded training, such as KnowBe4, Mimecast, Proofpoint (formerly Wombat), MediaPro, Cofense (formerly Phishme) and many others. Our work has the potential to contribute to improving such systems by accounting for individual difference characteristics when delivering and evaluating training.

### Conclusion

Online phishing has posed a large risk to both individual and organisational information security, with increasing popularity due to the flaw in human susceptibility [61]. Using a novel, current and reliable measure of phishing detection, we found relationships between several individual difference variables and both cognitive and behavioural indications of phishing susceptibility and false positives. Ultimately, our study has provided further insight into the key role of perceived maliciousness for detection accuracy, as well as the predictive role of individual difference variables and awareness of and competence about phishing and computers for discriminating malicious email. Variables examined accounted for a moderate to large amount of variance in both maliciousness perception and behavioural responses for both genuine and phishing email.

Following the current results, we suggest that future research on phishing susceptibility employs a methodology that allows susceptibility to be captured as a robust tendency instead of a one-off choice, thus allowing for the proper examination of individual differences.

## Supporting information

S1 Appendix “S1_Appendix.docx”. This document contains Fig A, Fig B and Fig C and Tables A, B and C. Fig A. This is the distribution of participant behaviour responses on percentages of participants. Fig B. This is the most effective phishing email from the PDT. Fig C. This is the genuine email with the most false positives in the PDT. Table A. This is a table with the examples of participant responses for defining what a phishing email is. Table B. This is the list of items in the Risk Profile Questionnaire. Table C. This is the results for the t-tests run on measured variables with phishing susceptibility and false positive variables.
(DOCX)
