# Supplementary material for: It’s the deceiver and the receiver: Individual differences in phishing susceptibility and false positives with item profiling
Source: PLoS One. 2018 Oct 26;13(10):e0205089. doi: 10.1371/journal.pone.0205089 (PMC6203253; doi:10.1371/journal.pone.0205089)
Supplement: S1 Appendix — This document contains Fig A, Fig B and Fig C and Tables A, B and C. Fig A. This is the distribution of participant behaviour responses on percentages of participants. Fig B. This is the most effective phishing email from the PDT. Fig C. This is the genuine email with the most false positives in the PDT. Table A. This is a table with the examples of participant responses for defining what a phishing email is. Table B. This is the list of items in the Risk Profile Questionnaire. Table C. This is the results for the t-tests run on measured variables with phishing susceptibility and false positive variables. (DOCX) [file pone.0205089.s001.docx]

**Supporting Information**

S1 Appendix


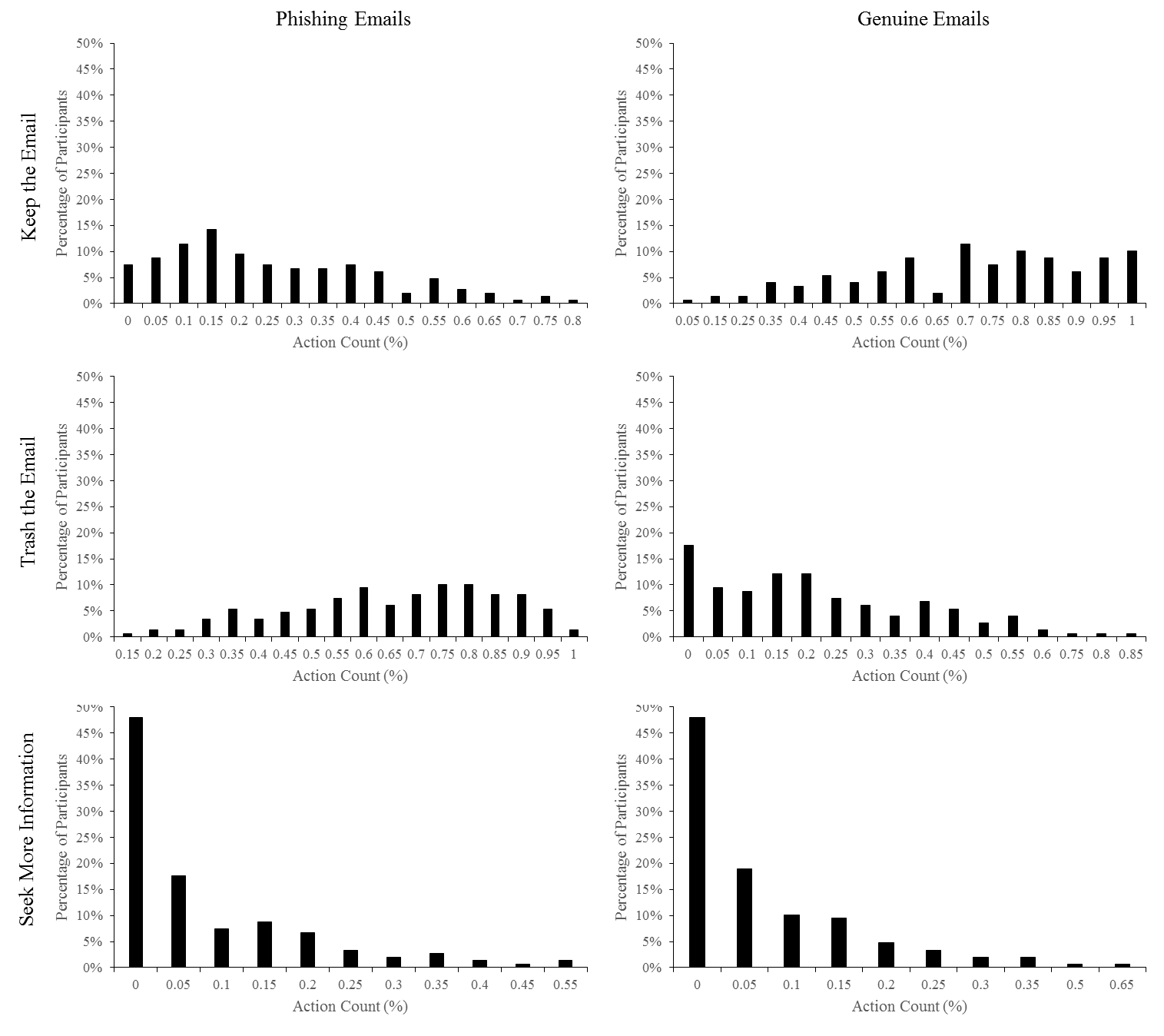


Fig A. Distribution of participant behaviour responses on percentages of participants. The six graphs correspond to the 3 behavioural responses (keeping the email, trashing the email, seeking more information) and the 2 types of email (phishing and genuine).


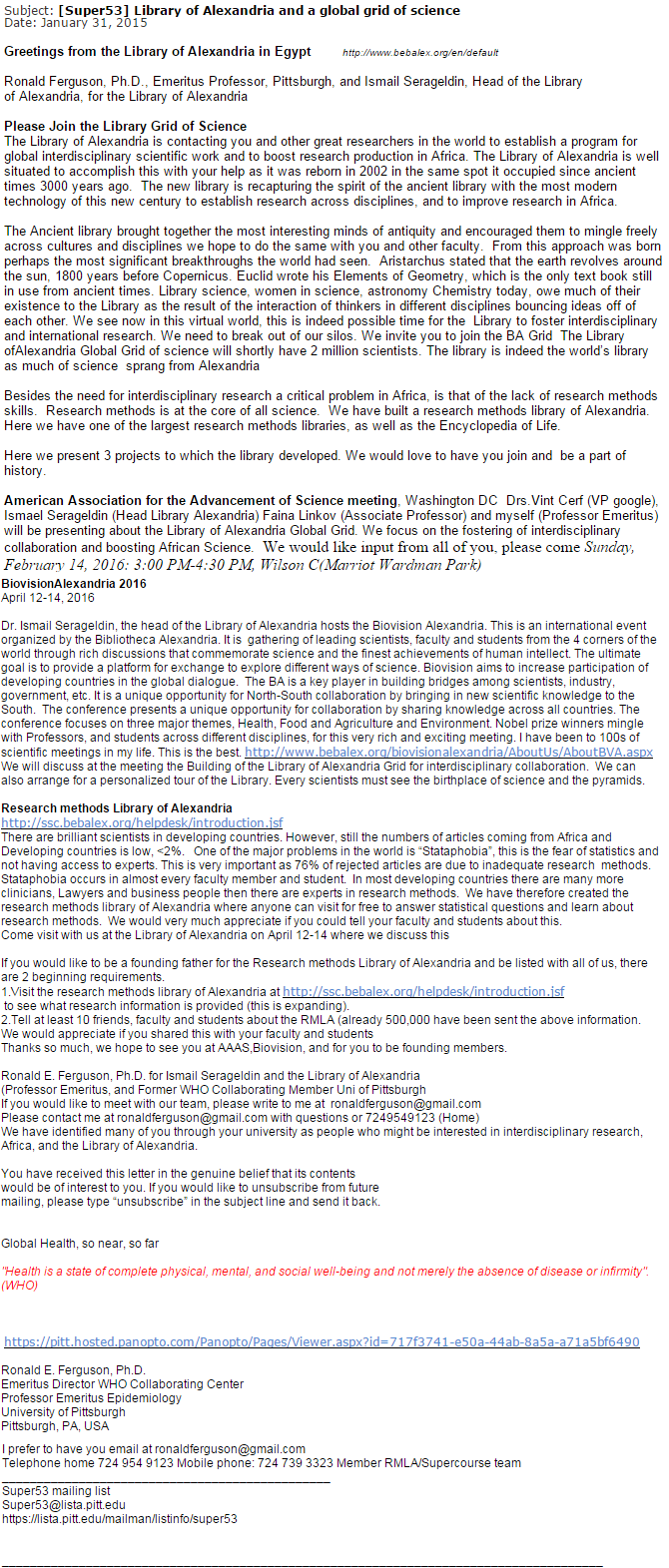


Fig B. The most trust-inducing phishing email in the PDT.


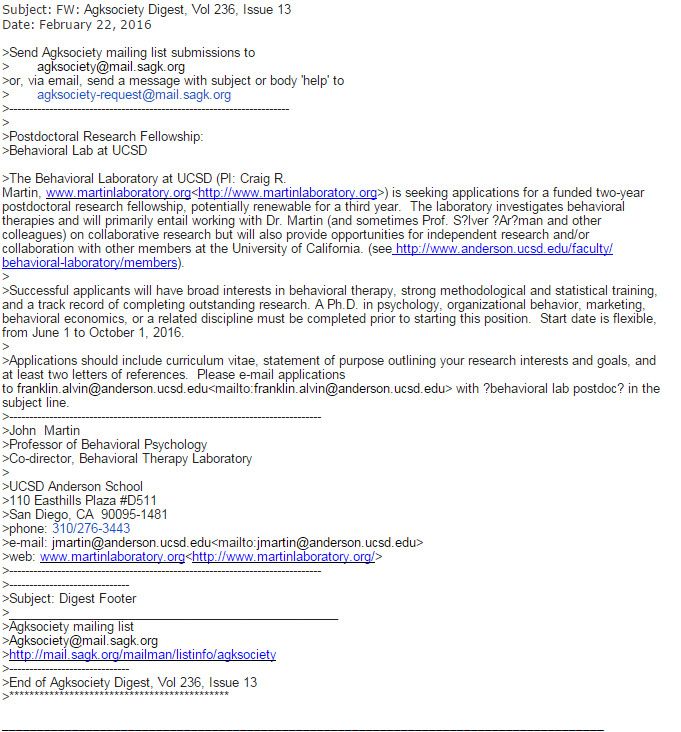


Fig C. The genuine email with highest frequency of being a false-positive in the PDT.

Table A.

Examples of Participant Responses to defining Phishing Email

| Average Rating | Definition |
| --- | --- |
| 3 | emails that try to scam the recipient into giving out their personal financial information under the  guise that the recipient will benefit from this, but in reality the "phishers" exploit this information for their own benef |
| 3 | deceptive emails which may try to access bank details under the guise of being another person or a legitimate company |
| 3 | Phishing email is a scamming method aimed at obtaining personal details by posing as a genuine email |
| 3 | A scam email that appears to be from a different source (hides true email) and usually asks for personal details/usernames or passwords |
| 2.67 | Emails with which the sender/s have the intention to reveal your personal information (credit card details, passwords to security accounts, etc). |
| 2.67 | An email that is a scam and attempts to trick you into giving out your personal information |
| 2.67 | fake email address of some famous companies to attract people to give personal details to make internet fraud. |
| 2.67 | The attempt to fool indiiduals throug email for monitary or other gain. e.g. 'A prince wants to give you money, just give your banking details'. |
| 2.67 | Email that is sent as spam normally through a virus that accesses and sends itself to people within someone's contact list,  in an attempt to trick people into revealing confidential information such as bank details. |
| 2.67 | Phishing email is a scam that attempts to gain your information, identity or access to your monetary funds for their own gain. |
| 2.67 | An email which is a scam in order to get the individuals personal detail such as bank accounts etc |
| 2.67 | Posing as someone else in an attempt to lure people into an online scam for their personal/financial details |
| 2.67 | Emails sent by hackers ridden with deceit in an attempt to obtain personal details such as credit card numbers |
| 2.67 | Trying to acquire personal information illegally by portraying a trustworthy company |
| 2.67 | emails that attempt to scam people into giving up personal information such as credit card details. They are scams |
| 2.67 | fraudulent emails from companies which attempt to get a person to reveal their personal details e.g. passwords. |
| 2.67 | emails from scammers that attempt to gain personal information about you such as credit card details and passwords. |
| 2.67 | Phishing scams are attempts by scammers to trick you into giving out personal information such as your  bank account numbers, passwords and credit card numbers. |
| 2.33 | An email that uses a fake alias and story in order to deceive the recipient out of money. |
| 2.33 | the illegal acquisition of data from people using malicious methods |
| 2.33 | A fake email in order to retrieve personal information from somebody by appealing to their emotions. |
| 2.33 | Email designed to trick the user into a malicious link or downloadable file. |
| 2.33 | Emails that deceive people into revealing personal details, e.g. bank account details |
| 2.33 | An email with the aim of deceiving an individual with the intention of a gain (usually financially) by the sender. |
| 2.33 | a fake email trying to scam someone in order for financial/other gain |
| 2.33 | Spam from someone/somewhere that's trying to get your personal details |
| 2.33 | an email that trys to deceive people in order to get benefit, especially money, from them |
| 2 | emails created to attempt to gather personal information |
| 2 | Email with the purpose of tricking you into being scammed. |
| 2 | It is a scam that attempts to steal personal information. |
| 2 | people trying to get hold of your personal information through misleading emails |
| 2 | emails from scammers that aim to gather your personal information |
| 2 | Email sent in an attempt to deceive one of their money |
| 2 | phone or online fake person to get your personal details such as bank details to cheat you |
| 2 | Trying to deceive the reading into providing personal information through an email |
| 2 | a malicious email used to gain financial information/password credentials etc. |
| 2 | when people try to obtain your personal details e.g. bank account details online |
| 2 | Email to get your details illegally and hack you computer |
| 2 | An email which is attempting to obtain personal information such as credit card number or password |
| 2 | Getting someone to give personal information via email |
| 2 | An email that attempts to gain personal information from the user |
| 2 | a scam that tries to get your personal details |
| 2 | Emails that attempt to gain information to scam people |
| 2 | when scammers try to take your PIN or other details to get your money |
| 2 | a fraudulent email that attempts to trick someone into agreeing to something they normally wouldn't |
| 2 | When scammers try to get your personal information, such as your password. |
| 2 | Gaining people's personal information through scams and spam emails |
| 2 | Scam to get personal information |
| 2 | attempt in requiring sensitive information |
| 2 | Phishing email is a scam email sent to gain specific information from someone. |
| 2 | Scam emails designed to get you to release personal information |
| 2 | scam emails trying to get personal information |
| 2 | Scammers use fishing emails to try and trick people into giving their personal details |
| 2 | Attempts by malacious entitites to obtain personal information to access what does not belong to them. |
| 2 | an email sent with the intent of scamming you out of money |
| 2 | An email with the intention of scamming or stealing personal information |
| 1.67 | Email that contain malcious content |
| 1.67 | Fake emails which trys to get personal details from users |
| 1.67 | A type of internet fraud through sending an email |
| 1.67 | an email intended to scam people for their money |
| 1.67 | emails that get personal information from you by tricking you |
| 1.67 | Email that tries to scam information or transmit a virus |
| 1.67 | A scam email that have a virus attached in an attempt to receive information from your computer |
| 1.67 | scammers trying to retrieve information |
| 1.67 | emails which attempt to trick you into something |
| 1.67 | scammers attempts to get your personal information |
| 1.67 | Email meant to make you open it with bad intent |
| 1.67 | email that attempts to acquire information illegally |
| 1.67 | Being manipulated to give personal information such as bank details |
| 1.67 | stealing personal information |
| 1.67 | attempting to acquire sensitive information via emailing |
| 1.67 | Emails that contain or contain links to viruses or scams |
| 1.67 | a scam email when they're trying to get money or information from you |
| 1.67 | Attempting to financially scam others |
| 1.33 | Emails that scam you by tricking the user into a desirable scenario |
| 1.33 | methods employed by individuals to scam others through the means of the internet |
| 1.33 | trying to hack into peoples emails, credit card accounts etc. |
| 1.33 | accessing others emails and taking information from without their knowledge |
| 1.33 | trick you into revealing info or clicking on link |
| 1.33 | Scam emails that make you give out personal details |
| 1.33 | fraudulent email |
| 1 | Scam emails |
| 1 | scam emails? |
| 1 | It's a dodgy email |
| 1 | online scams, usually done overseas |
| 1 | when they want to scam you |
| 1 | assume that it involves an attempted scam of some sort through rogue emails |
| 1 | An fake email is use to kite money |
| 1 | scamming emails |
| 1 | when someone tries to hack into/steal your email account |
| 1 | scam email |
| 1 | Scam email ? |
| 1 | fraud |
| 1 | scam email? |
| 1 | cyber crime |
| 1 | scam emails |
| 1 | when someone tries to take personal information |
| 1 | an email that is not from a legit source |
| 1 | A false email used for negative purposes? |
| 1 | scam email |
| 1 | form of fraud or scam |
| 0.67 | Malware used to lure users |
| 0.67 | scam |
| 0.67 | some kind of email |
| 0.67 | spam |
| 0.67 | used to try and scam people |
| 0.67 | Junk mail |
| 0.67 | Spam email |
| 0.67 | someone hacking your email |
| 0.67 | Sending a lot of emails to a large number of people |
| 0.33 | stealing data |
| 0.33 | emails that are ghjkhiohdjk |
| 0.33 | having access to sensitive information online - passwords |
| 0.33 | lure you into something you like, then replace that with something theyre trying to sell |
| 0 | I don't know |
| 0 | I have no idea |
| 0 | NA |
| 0 | I'm not sure |
| 0 | Not sure |
| 0 | Advertasment |
| 0 | i have no idea what it is |
| 0 | extra email used for reasons other than work and study |
| 0 | NA |
| 0 | I have no idea |
| 0 | i have no idea |
| 0 | no idea |
| 0 | Phishing emails are emails about fishing and boating |
| 0 | NA |
| 0 | An email where it can be interpreted in a number of different ways due to how it is said. |
| 0 | NA |
| 0 | NA |
| 0 | when you get rid of spam? |
| 0 | Phishing email is a type of email where you send informal emails |
| 0 | no idea |
| 0 | sorting through emails |
| 0 | email system that scans inbox for specific emails to categorise the inbox |
| 0 | copy |
| 0 | I don't know. Perhaps retrieving emails you have lost. |
| 0 | avoiding emails? |
| 0 | no idea |
| 0 | No clue |
| 0 | I have no idea what that means |
| 0 | No clue |
| 0 | I have no idea lol |
| 0 | communication through email |
| 0 | use some reward to induce the costumer to give out some benefits |
| 0 | cheat you |
| 0 | i have no idea |
| 0 | no idea, some sort of funky word |

Table B.

| Risk Profile Questionnaire Question List | |
| --- | --- |
| No. | Question |
| 1 | Have you ever noticed the "padlock" icon that appears in the lower right portion of your browser for certain websites? |
| 2 | Do you take measures to destroy old documents or materials with personal information prior to throwing them away? (Ex: shred or burn old checks, receipts, credit cards, etc.) |
| 3 | In your home or apartment, do you take measures to hide or protect valuable personal possessions (hiding place, safe, firebox, etc.)? |
| 4 | When informed by your computer system that a new update is available, do you usually choose to install the update? |
| 5 | Have you ever sought additional information to verify the legitimacy of an online retailer (checking Better Business Bureau, etc)? |
| 6 | Do you usually look for or read over a website's Privacy Policy before providing your confidential information (SS#, credit card #, bank account #, etc.)? |
| 7 | Have you ever stopped a transaction or avoid a transaction because you did not see a seal of approval such as Verisign listed at checkout? |

Table C.

**Two-sample t-test Significance Testing^[[1]](#footnote-1)^**

|  | | | | |  |
| --- | --- | --- | --- | --- | --- |
| **Independent Variable** | **Dependent Variable** | **Group 1 Mean** | **Group 2 Mean** | **t-value** | **p.value** |
| English as a First Language | Phishing Detection Accuracy | 0.69 | 0.76 | -1.91 | 0.06 |
| English as a First Language | Genuine Detection Accuracy | 0.73 | 0.80 | -2.03 | **0.04*** |
| English as a First Language | Likelihood of Maliciousness Phishing | 62.73 | 67.72 | -1.86 | 0.06 |
| English as a First Language | Likelihood of Maliciousness Genuine | 33.44 | 25.89 | 2.61 | **0.01*** |
| Gender | Phishing Detection Accuracy | 0.75 | 0.71 | 1.21 | 0.23 |
| Gender | Genuine Detection Accuracy | 0.79 | 0.77 | 0.76 | 0.45 |
| Gender | Likelihood of Maliciousness Phishing | 65.14 | 68.98 | -1.54 | 0.12 |
| Gender | Likelihood of Maliciousness Genuine | 27.02 | 29.41 | -0.88 | 0.38 |
| Living in Australia Prior to University | Phishing Detection Accuracy | 0.74 | 0.74 | 0.07 | 0.94 |
| Living in Australia Prior to University | Genuine Detection Accuracy | 0.74 | 0.79 | -0.87 | 0.39 |
| Living in Australia Prior to University | Likelihood of Maliciousness Phishing | 69.18 | 66.17 | 0.72 | 0.47 |
| Living in Australia Prior to University | Likelihood of Maliciousness Genuine | 37.37 | 26.91 | 2.33 | **0.02*** |
| Risk Profile Questionnaire Q1 | Phishing Detection Accuracy | 0.70 | 0.77 | -2.16 | **0.03*** |
| Risk Profile Questionnaire Q1 | Genuine Detection Accuracy | 0.79 | 0.78 | 0.36 | 0.72 |
| Risk Profile Questionnaire Q1 | Likelihood of Maliciousness Phishing | 64.12 | 68.10 | -1.67 | 0.10 |
| Risk Profile Questionnaire Q1 | Likelihood of Maliciousness Genuine | 28.94 | 27.02 | 0.73 | 0.46 |
| Risk Profile Questionnaire Q2 | Phishing Detection Accuracy | 0.73 | 0.74 | -0.40 | 0.69 |
| Risk Profile Questionnaire Q2 | Genuine Detection Accuracy | 0.80 | 0.77 | 0.79 | 0.43 |
| Risk Profile Questionnaire Q2 | Likelihood of Maliciousness Phishing | 64.69 | 67.77 | -1.29 | 0.20 |
| Risk Profile Questionnaire Q2 | Likelihood of Maliciousness Genuine | 26.89 | 28.54 | -0.63 | 0.53 |
| Risk Profile Questionnaire Q3 | Phishing Detection Accuracy | 0.78 | 0.72 | 1.91 | 0.06 |
| Risk Profile Questionnaire Q3 | Genuine Detection Accuracy | 0.78 | 0.78 | -0.19 | 0.85 |
| Risk Profile Questionnaire Q3 | Likelihood of Maliciousness Phishing | 68.87 | 65.27 | 1.44 | 0.15 |
| Risk Profile Questionnaire Q3 | Likelihood of Maliciousness Genuine | 30.30 | 26.64 | 1.34 | 0.18 |
| Risk Profile Questionnaire Q4 | Phishing Detection Accuracy | 0.75 | 0.73 | 0.58 | 0.56 |
| Risk Profile Questionnaire Q4 | Genuine Detection Accuracy | 0.81 | 0.75 | 1.81 | 0.07 |
| Risk Profile Questionnaire Q4 | Likelihood of Maliciousness Phishing | 67.21 | 65.70 | 0.64 | 0.52 |
| Risk Profile Questionnaire Q4 | Likelihood of Maliciousness Genuine | 25.86 | 29.69 | -1.49 | 0.14 |
| Risk Profile Questionnaire Q5 | Phishing Detection Accuracy | 0.71 | 0.77 | -1.83 | 0.07 |
| Risk Profile Questionnaire Q5 | Genuine Detection Accuracy | 0.79 | 0.77 | 0.59 | 0.55 |
| Risk Profile Questionnaire Q5 | Likelihood of Maliciousness Phishing | 64.27 | 68.60 | -1.85 | 0.07 |
| Risk Profile Questionnaire Q5 | Likelihood of Maliciousness Genuine | 25.48 | 30.18 | -1.84 | 0.07 |
| Risk Profile Questionnaire Q6 | Phishing Detection Accuracy | 0.75 | 0.72 | 1.09 | 0.28 |
| Risk Profile Questionnaire Q6 | Genuine Detection Accuracy | 0.79 | 0.77 | 0.66 | 0.51 |
| Risk Profile Questionnaire Q6 | Likelihood of Maliciousness Phishing | 66.74 | 65.92 | 0.33 | 0.74 |
| Risk Profile Questionnaire Q6 | Likelihood of Maliciousness Genuine | 26.47 | 30.12 | -1.38 | 0.17 |
| Risk Profile Questionnaire Q7 | Phishing Detection Accuracy | 0.73 | 0.75 | -0.52 | 0.61 |
| Risk Profile Questionnaire Q7 | Genuine Detection Accuracy | 0.80 | 0.76 | 1.26 | 0.21 |
| Risk Profile Questionnaire Q7 | Likelihood of Maliciousness Phishing | 66.20 | 66.83 | -0.26 | 0.80 |
| Risk Profile Questionnaire Q7 | Likelihood of Maliciousness Genuine | 26.73 | 29.68 | -1.11 | 0.27 |
| * p< .05 |  |  |  |  |  |

1. English as a First Language Groups (0 = No, 1 = Yes); Gender Groups (0 = Female, 1 = Male); Living in Australia Prior to University Groups (0 = No, 1 = Yes); Although there was a significant difference between those who had lived in Australia prior to university and those who did not, there were only 13 participants in the latter group, restricting the needed statistical power for meaningful analyses [↑](#footnote-ref-1)
